# Identification and Functional Characterization of *G6PC2* Coding Variants Influencing Glycemic Traits Define an Effector Transcript at the *G6PC2-ABCB11* Locus

**DOI:** 10.1371/journal.pgen.1004876

**Published:** 2015-01-27

**Authors:** Anubha Mahajan, Xueling Sim, Hui Jin Ng, Alisa Manning, Manuel A. Rivas, Heather M. Highland, Adam E. Locke, Niels Grarup, Hae Kyung Im, Pablo Cingolani, Jason Flannick, Pierre Fontanillas, Christian Fuchsberger, Kyle J. Gaulton, Tanya M. Teslovich, N. William Rayner, Neil R. Robertson, Nicola L. Beer, Jana K. Rundle, Jette Bork-Jensen, Claes Ladenvall, Christine Blancher, David Buck, Gemma Buck, Noël P. Burtt, Stacey Gabriel, Anette P. Gjesing, Christopher J. Groves, Mette Hollensted, Jeroen R. Huyghe, Anne U. Jackson, Goo Jun, Johanne Marie Justesen, Massimo Mangino, Jacquelyn Murphy, Matt Neville, Robert Onofrio, Kerrin S. Small, Heather M. Stringham, Ann-Christine Syvänen, Joseph Trakalo, Goncalo Abecasis, Graeme I. Bell, John Blangero, Nancy J. Cox, Ravindranath Duggirala, Craig L. Hanis, Mark Seielstad, James G. Wilson, Cramer Christensen, Ivan Brandslund, Rainer Rauramaa, Gabriela L. Surdulescu, Alex S. F. Doney, Lars Lannfelt, Allan Linneberg, Bo Isomaa, Tiinamaija Tuomi, Marit E. Jørgensen, Torben Jørgensen, Johanna Kuusisto, Matti Uusitupa, Veikko Salomaa, Timothy D. Spector, Andrew D. Morris, Colin N. A. Palmer, Francis S. Collins, Karen L. Mohlke, Richard N. Bergman, Erik Ingelsson, Lars Lind, Jaakko Tuomilehto, Torben Hansen, Richard M. Watanabe, Inga Prokopenko, Josee Dupuis, Fredrik Karpe, Leif Groop, Markku Laakso, Oluf Pedersen, Jose C. Florez, Andrew P. Morris, David Altshuler, James B. Meigs, Michael Boehnke, Mark I. McCarthy, Cecilia M. Lindgren, Anna L. Gloyn, On Behalf of the T2D-GENES consortium and GoT2D consortium

**Affiliations:** 1 Wellcome Trust Centre for Human Genetics, Nuffield Department of Medicine, University of Oxford, Oxford, United Kingdom; 2 Department of Biostatistics and Center for Statistical Genetics, University of Michigan, Ann Arbor, Michigan, United States of America; 3 Oxford Centre for Diabetes, Endocrinology and Metabolism, Radcliffe Department of Medicine, University of Oxford, Oxford, United Kingdom; 4 Program in Medical and Population Genetics, Broad Institute, Cambridge, Massachusetts, United States of America; 5 Human Genetics Center, The University of Texas Graduate School of Biomedical Sciences at Houston, The University of Texas Health Science Center at Houston, Houston, Texas, United States of America; 6 The Novo Nordisk Foundation Center for Basic Metabolic Research, Faculty of Health and Medical Sciences, University of Copenhagen, Copenhagen, Denmark; 7 Department of Health Studies, Biostatistics Laboratory, The University of Chicago, Chicago, Illinois, United States of America; 8 School of Computer Science, McGill University, Montreal, Quebec, Canada; 9 McGill University and Génome Québec Innovation Centre, Montreal, Quebec, Canada; 10 Department of Molecular Biology, Massachusetts General Hospital, Boston, Massachusetts, United States of America; 11 Department of Human Genetics, Wellcome Trust Sanger Institute, Hinxton, Cambridgeshire, United Kingdom; 12 Department of Clinical Sciences, Diabetes and Endocrinology, Lund University Diabetes Centre, Malmö, Sweden; 13 High Throughput Genomics, Oxford Genomics Centre, Wellcome Trust Centre for Human Genetics, Nuffield Department of Medicine, University of Oxford, Oxford, United Kingdom; 14 Department of Twin Research and Genetic Epidemiology, King’s College London, London, United Kingdom; 15 Department of Medical Sciences, Molecular Medicine and Science for Life Laboratory, Uppsala University, Uppsala, Sweden; 16 Departments of Medicine and Human Genetics, The University of Chicago, Chicago, Illinois, United States of America; 17 Department of Genetics, Texas Biomedical Research Institute, San Antonio, Texas, United States of America; 18 Department of Medicine, Section of Genetic Medicine, The University of Chicago, Chicago, Illinois, United States of America; 19 Human Genetics Center, School of Public Health, The University of Texas Health Science Center at Houston, Houston, Texas, United States of America; 20 Blood Systems Research Institute, San Francisco, California, United States of America; 21 Department of Laboratory Medicine & Institute for Human Genetics, University of California, San Francisco, San Francisco, California, United States of America; 22 Department of Physiology and Biophysics, University of Mississippi Medical Center, Jackson, Mississippi, United States of America; 23 Department of Internal Medicine and Endocrinology, Vejle Hospital, Vejle, Denmark; 24 Department of Clinical Biochemistry, Vejle Hospital, Vejle, Denmark; 25 Institute of Regional Health Research, University of Southern Denmark, Odense, Denmark; 26 Foundation for Research in Health, Exercise and Nutrition, Kuopio Research Institute of Exercise Medicine, Kuopio, Finland; 27 Division of Cardiovascular and Diabetes Medicine, Medical Research Institute, Ninewells Hospital and Medical School, Dundee, United Kingdom; 28 Department of Public Health and Caring Sciences, Geriatrics, Uppsala University, Uppsala, Sweden; 29 Department of Clinical Experimental Research, Glostrup University Hospital, Glostrup, Denmark; 30 Department of Clinical Medicine, Faculty of Health and Medical Sciences, University of Copenhagen, Copenhagen, Denmark; 31 Research Centre for Prevention and Health, Glostrup University Hospital, Glostrup, Denmark; 32 Department of Social Services and Health Care, Jakobstad, Finland; 33 Folkhälsan Research Centre, Helsinki, Finland; 34 Department of Endocrinology, Helsinki University Central Hospital, Helsinki, Finland; 35 Steno Diabetes Center, Gentofte, Denmark; 36 Faculty of Medicine, University of Aalborg, Aalborg, Denmark; 37 Faculty of Health Sciences, Institute of Clinical Medicine, Internal Medicine, University of Eastern Finland, Kuopio, Finland; 38 Kuopio University Hospital, Kuopio, Finland; 39 Institute of Public Health and Clinical Nutrition, University of Eastern Finland, Kuopio, Finland; 40 National Institute for Health and Welfare, Helsinki, Finland; 41 Clinical Research Centre, Centre for Molecular Medicine, Ninewells Hospital and Medical School, Dundee, United Kingdom; 42 Pat Macpherson Centre for Pharmacogenetics and Pharmacogenomics, Medical Research Institute, Ninewells Hospital and Medical School, Dundee, United Kingdom; 43 Medical Genomics and Metabolic Genetics Branch, National Human Genome Research Institute, National Institutes of Health, Bethesda, Maryland, United States of America; 44 Department of Genetics, University of North Carolina at Chapel Hill, Chapel Hill, North Carolina, United States of America; 45 Cedars-Sinai Diabetes and Obesity Research Institute, Los Angeles, California, United States of America; 46 Department of Medical Sciences, Molecular Epidemiology and Science for Life Laboratory, Uppsala University, Uppsala, Sweden; 47 Department of Medical Sciences, Uppsala University, Uppsala, Sweden; 48 Diabetes Research Group, King Abdulaziz University, Jeddah, Saudi Arabia; 49 Instituto de Investigacion Sanitaria del Hospital Universario LaPaz (IdiPAZ), University Hospital LaPaz, Autonomous University of Madrid, Madrid, Spain; 50 Center for Vascular Prevention, Danube University Krems, Krems, Austria; 51 Diabetes Prevention Unit, National Institute for Health and Welfare, Helsinki, Finland; 52 Faculty of Health Sciences, University of Southern Denmark, Odense, Denmark; 53 Department of Physiology & Biophysics, Keck School of Medicine, University of Southern California, Los Angeles, California, United States of America; 54 Department of Preventive Medicine, Keck School of Medicine, University of Southern California, Los Angeles, California, United States of America; 55 Diabetes and Obesity Research Institute, Keck School of Medicine, University of Southern California, Los Angeles, California, United States of America; 56 Department of Genomics of Common Disease, School of Public Health, Imperial College London, London, United Kingdom; 57 National Heart, Lung, and Blood Institute’s Framingham Heart Study, Framingham, Massachusetts, United States of America; 58 Department of Biostatistics, Boston University School of Public Health, Boston, Massachusetts, United States of America; 59 Oxford NIHR Biomedical Research Centre, Oxford University Hospitals Trust, Oxford, United Kingdom; 60 Diabetes Research Center (Diabetes Unit), Department of Medicine, Massachusetts General Hospital, Boston, Massachusetts, United States of America; 61 Department of Medicine, Harvard Medical School, Boston, Massachusetts, United States of America; 62 Center for Human Genetic Research, Department of Medicine, Massachusetts General Hospital, Boston, Massachusetts, United States of America; 63 Department of Biostatistics, University of Liverpool, Liverpool, United Kingdom; 64 Estonian Genome Centre, University of Tartu, Tartu, Estonia; 65 Department of Biology, Massachusetts Institute of Technology, Cambridge, Massachusetts, United States of America; 66 Department of Genetics, Harvard Medical School, Boston, Massachusetts, United States of America; 67 General Medicine Division, Massachusetts General Hospital and Department of Medicine, Harvard Medical School, Boston, Massachusetts, United States of America; University of Leicester, UNITED KINGDOM

## Abstract

Genome wide association studies (GWAS) for fasting glucose (FG) and insulin (FI) have identified common variant signals which explain 4.8% and 1.2% of trait variance, respectively. It is hypothesized that low-frequency and rare variants could contribute substantially to unexplained genetic variance. To test this, we analyzed exome-array data from up to 33,231 non-diabetic individuals of European ancestry. We found exome-wide significant (P<5×10-7) evidence for two loci not previously highlighted by common variant GWAS: *GLP1R* (p.Ala316Thr, minor allele frequency (MAF)=1.5%) influencing FG levels, and *URB2* (p.Glu594Val, MAF = 0.1%) influencing FI levels. Coding variant associations can highlight potential effector genes at (non-coding) GWAS signals. At the *G6PC2/ABCB11* locus, we identified multiple coding variants in *G6PC2* (p.Val219Leu, p.His177Tyr, and p.Tyr207Ser) influencing FG levels, conditionally independent of each other and the non-coding GWAS signal. *In vitro* assays demonstrate that these associated coding alleles result in reduced protein abundance via proteasomal degradation, establishing *G6PC2* as an effector gene at this locus. Reconciliation of single-variant associations and functional effects was only possible when haplotype phase was considered. In contrast to earlier reports suggesting that, paradoxically, glucose-raising alleles at this locus are protective against type 2 diabetes (T2D), the p.Val219Leu *G6PC2* variant displayed a modest but directionally consistent association with T2D risk. Coding variant associations for glycemic traits in GWAS signals highlight *PCSK1*, *RREB1*, and *ZHX3* as likely effector transcripts. These coding variant association signals do not have a major impact on the trait variance explained, but they do provide valuable biological insights.

## Author Summary

Understanding how FI and FG levels are regulated is important because their derangement is a feature of T2D. Despite recent success from GWAS in identifying regions of the genome influencing glycemic traits, collectively these loci explain only a small proportion of trait variance. Unlocking the biological mechanisms driving these associations has been challenging because the vast majority of variants map to non-coding sequence, and the genes through which they exert their impact are largely unknown. In the current study, we sought to increase our understanding of the physiological pathways influencing both traits using exome-array genotyping in up to 33,231 non-diabetic individuals to identify coding variants and consequently genes associated with either FG or FI levels. We identified novel association signals for both traits including the receptor for GLP-1 agonists which are a widely used therapy for T2D. Furthermore, we identified coding variants at several GWAS loci which point to the genes underlying these association signals. Importantly, we found that multiple coding variants in *G6PC2* result in a loss of protein function and lower fasting glucose levels.

## Introduction

Large-scale GWAS of non-diabetic individuals have successfully identified > 60 loci associated with FG and FI levels, many of which are also implicated in susceptibility to T2D [1, 2, 3, 4]. Despite these successes, lead SNPs at GWAS loci have modest effects and cumulatively explain only a small proportion of the trait variance in non-diabetic individuals. By design, GWAS have focused predominantly on the interrogation of common variants, defined here to have MAF > 5%. Most of the identified variants are non-coding, complicating attempts to establish the molecular consequences of these GWAS loci. We therefore chose to extend discovery efforts to coding variants, particularly those of lower frequency that have not been well captured by GWAS genotyping and imputation. We aimed both to identify novel coding loci for FG and FI, and to evaluate the role of coding variants at known GWAS loci, thereby expecting to highlight causal transcripts and to facilitate characterization of the molecular mechanisms influencing glycemic traits and T2D susceptibility.

## Results

We analyzed 33,231 (FG) and 30,825 (FI) non-diabetic individuals from 14 studies of European ancestry, all genotyped with the Illumina HumanExome BeadChip (**see URLs**). Characteristics of the contributing studies and study participants are summarized in S1-S2 Tables. Body mass index (BMI) adjustment has been shown to increase power to detect association with these glycemic traits [4], and in our study samples, BMI accounted for 6.1% and 24.6% of phenotypic variance of FG and FI, respectively. Consequently, within each study, we calculated residuals for both traits after adjustment for BMI and other study-specific covariates (S1 Table). Study-specific inverse-rank normalized residuals were tested for single-variant association using a linear mixed model to account for relatedness and fine-scale genetic population sub-structure [5]. We also repeated the analysis using the untransformed residuals to obtain allelic effect sizes. We then combined the association summary statistics across studies using fixed-effect meta-analysis. We restricted our single-variant analysis to 106,489 variants that pass quality-control and are polymorphic in more than one study. We declared a single-variant trait association as exome-wide significant at *P* < 5×10^-7^, corresponding to Bonferroni correction for the ~100,000 polymorphic variants. We also carried out gene-based meta-analysis [6, 7] by using the sequence kernel association test (SKAT) [8] and a frequency-weighted burden test [9] applying four alternate variant masks which combine functional annotation and allele frequency thresholds. Full details of the variant masks are provided in the Methods. Gene-based tests take into account overall variant-load within a specified locus and therefore may have greater power than single-variant tests to detect associations with multiple rare and low-frequency causal alleles. We note that this advantage is likely to be less in exome-array analysis compared with the more complete ascertainment of variants possible with exome sequencing [10]. We declared gene-based association as exome-wide significant at *P* < 2.5×10^-6^, corresponding to Bonferroni correction for ~20,000 protein-coding genes in the genome.

### Coding variants influencing FG levels

Through single-variant analysis, we identified 12 coding variants (all of which were non-synonymous changes) associated with FG levels at exome-wide significance, two low-frequency and ten common (S3 Table). These variants mapped to seven loci, six of them previously implicated in FG regulation. The signals at known loci included previously-reported common coding variants driving GWAS signals at *GCKR* (p.Pro446Leu, MAF = 36.9%, *P* = 5.3×10^−18^) and *SLC30A8* (p.Arg325Trp, MAF = 35.7%, *P* = 2.5×10^−10^) [2, 11]. Three additional common coding variants associated with FG (in *C2orf16*, *GPN1*, and *SLC5A6*) were in moderate linkage disequilibrium (LD; *r^2^* = 0.2–0.4) with the p.Pro446Leu *GCKR* variant. Their associations were eliminated after conditioning on p.Pro446Leu, the known functional GWAS variant in this region [12, 13], indicating no causal role for these additional variants on FG regulation (S3 Table).

The sixth variant influencing FG levels at exome-wide significance was a low-frequency non-synonymous change which did not map to any previously known FG-influencing locus: p.Ala316Thr at *GLP1R* (MAF = 1.5%, *P* = 4.6×10^−7^; Table 1 and S1B Fig.). This variant showed modest association (*P* = 1.3×10^−4^) in a previous GWAS meta-analysis of FG [3], which partially overlaps with the present study, but now achieves exome-wide significance. The alanine residue at p.Ala316Thr is conserved across vertebrates, and the threonine substitution is predicted to be “possibly damaging” by *in silico* mutation analysis (S4 Table). GLP-1R (glucagon-like peptide 1 receptor) is the receptor for the incretin hormone glucagon-like peptide 1 (GLP1), which is released from enteroendocrine cells after food ingestion and potentiates insulin secretion. GLP1 receptor agonists are an established treatment for T2D [14].

**Table 1:** Coding variants associated with FG and FI levels at exome-wide significance.

**SNP**	**Gene**	**Chr**	**Variant**	**Minor/Major allele^a^**	**MAF^b^ (%)**	**N**	**l^2^**	**Cochran’s Q**	***P*_het**	**Unconditional**		**Conditional**	
										***P^c^***	$\hat{\beta }$ **(SE)**	***P***	$\hat{\beta }$ **(SE)**
**Fasting glucose**													
rs138726309	*G6PC2*	2	p.His177Tyr	T/C	0.8	32,430	27.2	16.49	0.17	3.1×10^-8^	-0.102 (0.020)	^d^ 1.3×10^−11^	-0.125 (0.020)
												^e^ 3.5×10^−10^	-0.115 (0.020)
rs492594	*G6PC2*	2	p.Val219Leu	C/G	48.1	33,231	0	6.68	0.92	6.0×10^-9^	0.020 (0.004)	^d^ 7.1×10^−10^	-0.034 (0.005)
												^f^ 6.5×10^−13^	-0.032 (0.005)
rs6234	*PCSK1*	5	p.Gln665Glu	C/G	27.9	33,231	0	8.58	0.80	3.0×10^-8^	-0.022 (0.004)	-	-
rs6235	*PCSK1*	5	p.Ser690Thr	G/C	27.9	33,231	0	8.65	0.80	4.1×10^-8^	-0.022 (0.004)	-	-
rs35742417	*RREB1*	6	p.Ser1554Tyr	A/C	21.1	33,230	0	12.28	0.51	8.4×10^-9^	-0.024 (0.004)	-	-
rs10305492	*GLP1R*	6	p.Ala316Thr	A/G	1.5	33,230	0	11.29	0.59	4.6×10^-7^	-0.073 (0.015)	-	-
rs17265513	*ZHX3*	20	p.Asn310Ser	C/T	23.8	33,229	0	12.01	0.53	3.9×10^-7^	0.022 (0.004)	-	-
**Fasting insulin**													
rs141203811	*URB2*	1	p.Glu594Val	T/A	0.1	21,130	59.9	9.98	0.04	3.1×10^-7^	0.282 (0.066)	-	-

Chr: chromosome. MAF: minor allele frequency. N: number of samples analyzed. I^2^: heterogeneity measure in %. *P*_het: P-value for Cochran’s Q statistic. $\hat{\beta }$: regression coefficient estimates. SE: standard error.

^a^Alleles are aligned to the forward strand of NCBI Build 37.

^b^Sample-size weighted average minor allele frequency percentage across all studies.

^c^Sample-size Z-score weighted *P* values are obtained with derived inverse normalized residuals of mmol/L of fasting glucose and pmol/L of natural log-transformed fasting insulin after adjustment for age, sex, and BMI. Effect size estimates are reported for the minor allele in mmol/L of fasting glucose and pmol/L of natural log-transformed fasting insulin after adjustment for age, sex, and BMI.

^d^After adjusting for the common lead non-coding GWAS SNP rs560887.

^e^After adjusting for the common lead non-coding GWAS SNP rs560887 and the common non-synonymous variant p.Val219Leu.

^f^After adjusting for the common lead non-coding GWAS SNP rs560887 and the low-frequency non-synonymous variant p.His177Tyr.

The six remaining coding variants all map to known FG-associated GWAS loci, but have not previously been implicated as playing a causal role. These include two variants in the islet-specific glucose-6-phosphatase catalytic subunit (*G6PC2*) gene at the *G6PC2*/*ABCB11* locus: p.Val219Leu, a common variant (*P* = 6.0×10^−9^, MAF = 48.1%), and p.His177Tyr, a low-frequency variant (*P* = 3.1×10^−8^, MAF = 0.8%; S2A Fig.). These variants remained significantly associated (p.Val219Leu, *P_conditional_* = 7.1×10^−10^ and p.His177Tyr, *P_conditional_* = 1.3×10^−11^) with FG after conditioning on the intronic lead GWAS SNP, rs560887 (Table 1 and S2B Fig.). Conversely, conditioning on the coding variants did not completely abolish the association signal at the lead GWAS SNP (*P*_unconditional_ = 6.4×10^−78^; *P_conditional on_*_His177Tyr_ = 3.1×10^−55^, *P_conditional on_*_Val219Leu_ = 1.2×10^−58^, and *P_conditional on_*_His177Tyr and Val219Leu_ = 2.1×10^−83^), confirming that the effect of the coding variants were largely independent of the lead GWAS SNP. Furthermore, the coding variants each remained associated with FG at exome-wide significance even after conditioning on both the lead GWAS SNP and the other coding variant, providing clear evidence of at least three association signals at this locus (Table 1 and S2C-S2D Fig.). These results are consistent with a recent study in Finnish individuals that reported a FG association signal at p.His177Tyr [15], but we extend that finding by demonstrating exome-wide significant association of multiple coding variants after conditioning on other associated variants in the region.

*G6PC2* was also the only one of the 14,465 genes with multiple exome-array variants to demonstrate evidence of significant association with FG levels with any mask in gene-based tests (Table 2). In fact, for this gene we observed significant association with FG levels for all the masks encompassing multiple variants. These gene-based associations remained significant even after Bonferroni correction for testing of four different variant masks. Step-wise conditional analyses, adjusting for each variant included in the respective masks, revealed that the gene-based signals were primarily driven by two variants: p.His177Tyr and p.Tyr207Ser (S5 Table). The protein-truncating variant (PTV) p.Arg283*, which introduces a stop mutation in the last exon of the gene, showed no association with FG levels in single-variant or gene-based analyses. The most likely explanation is that this variant evades nonsense mediated decay (as is usual for PTV in the last exon) and that the truncated protein (missing only the terminal 72 amino acid residues) retains normal functional activity [16]. Due to its high allele frequency, and annotation as benign across multiple annotation algorithms, p.Val219Leu was not included in any of the gene-based variant masks (S6 Table). However, based on the single-variant it also has independent effects, with the result that we have three coding variants in *G6PC2* (p.His177Tyr, p.Tyr207Ser, and p.Val219Leu) influencing FG levels. These three variants explain an additional 0.2% of the phenotypic variance in FG beyond that explained by the GWAS variant rs560887, bringing the total variance explained by this locus to 1.1%. However, we recognize that this estimate has been obtained in the discovery cohort and consequently might be inflated.

**Table 2:** *G6PC2* gene-based association with FG levels using SKAT and BURDEN test

**Mask**	**Number of variants**	**Average allele frequency (%)**	***P*_SKAT_**	***P*_BURDEN_**
**Mask 1**: PTV-only	0	-	-	-
**Mask 2**: PTV + missense	15	0.10	1.8×10^-13^	4.1×10^-16^
**Mask 3**: PTV + NS_strict_	4	0.28	3.6×10^-12^	5.1×10^-13^
**Mask 4**: PTV + NS_broad_	12	0.12	2.0×10^-13^	1.2×10^-17^

PTV: protein-truncating variant; NS: non-synonymous variants; NS_strict_: missense variant predicted to be deleterious by all five annotation algorithms (Polyphen2-HumDiv, PolyPhen2-HumVar, LRT, MutationTaster, and SIFT); NS_broad_: missense variant predicted to be deleterious by at least one of the five annotation algorithms.

Mask 1: “PTV-only” encompassed only one variant.

Mask 2 consists of PTVs and all missense variants with MAF<1%; Mask 3 consists of PTVs and missense variants predicted to be deleterious by five annotation algorithms without any upper MAF bounds; Mask 4 consists of variants in Mask 3 plus any missense variant with MAF<1% predicted to be deleterious by at least one of the five annotation algorithms.

Allele counts of the 15 non-synonymous variants at *G6PC2*: p.Ser324Pro (56), p.Leu310Phe (42), p.Arg283* (71), pIle273Val (2), p.Phe256Leu (9), p.Ile230Thr (14), p. Tyr207Ser (338), p.His177Tyr (502), p.Ile171Thr (81), p.Ile171Val (4), p.Ala119Val (23), p.Asn68Ile (2), p.Ile63Thr (6), p.Ile38Leu (1), p.Ser30Phe (23).

Both *G6PC2* and *ABCB11* have been considered strong biological candidate genes for glucose regulation, and advocated as potential effector transcripts at this GWAS locus [17, 18]. However, none of the 21 coding variants in *ABCB11* that passed quality control (twelve rare, eight low-frequency, and one common) were significantly associated with FG levels, nor was there an aggregate association signal (*P*>0.05). Together, our genetic data provide compelling evidence that *G6PC2* is an effector gene for FG regulation at this GWAS locus, with p.His177Tyr, p.Tyr207Ser, and p.Val219Leu as likely causal coding variants.

### Loss of G6PC2 function leads to a reduction in FG levels

**Figure 1:**
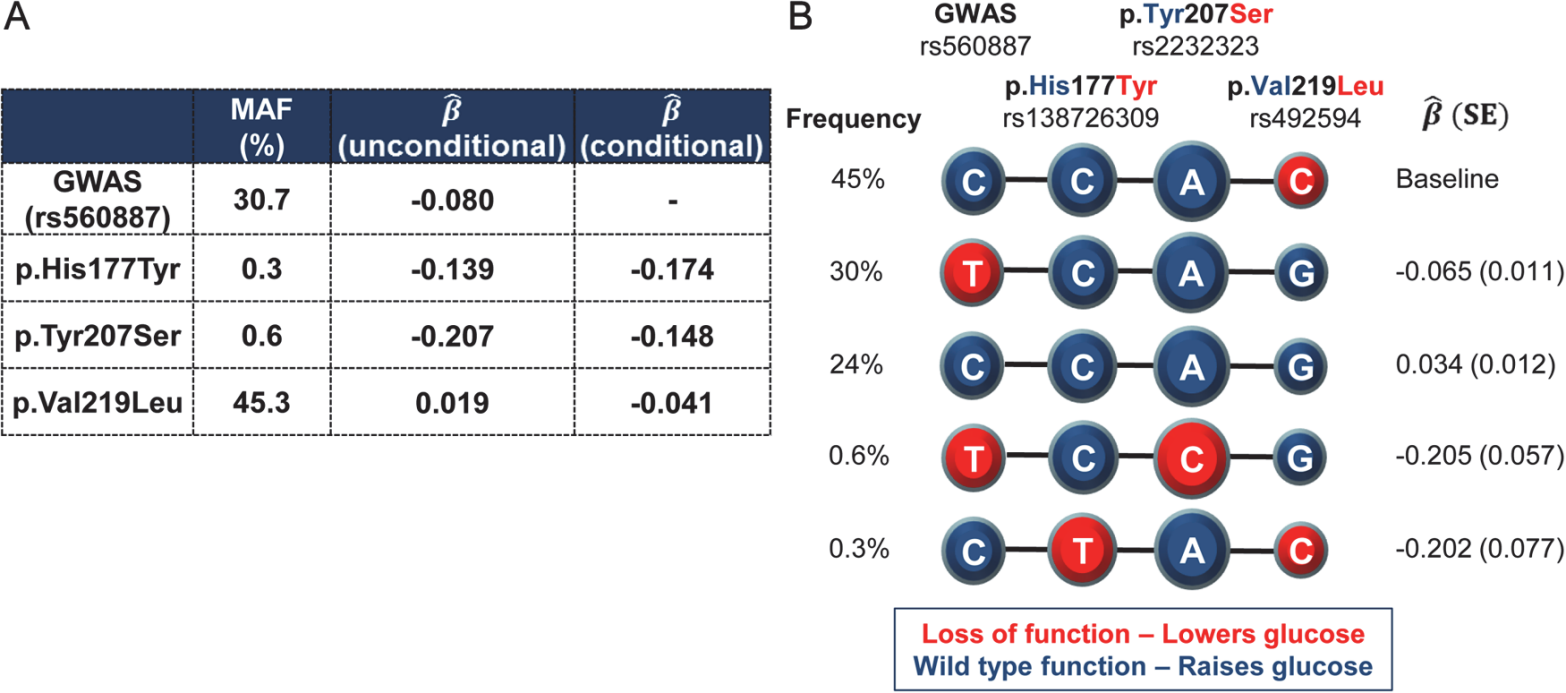
Haplotypes of the lead non-coding GWAS SNP rs560887 and the three coding variants. rs138726309 (p.His177Tyr), rs2232323 (p.Tyr207Ser), and rs492594 (p.Val219Leu), obtained from 4,442 unrelated individuals from the Oxford Biobank. (A) Percentage minor allele frequency (MAF) and effect size estimates ($\hat{\beta }$) of the four variants reported for the minor allele in mmol/L of FG after adjustment for age, sex, and BMI. (B) Haplotypes of the four associated variants in G6PC2 revealed that the glucose-lowering Leu219 allele was carried exclusively in cis with the glucose-raising allele at the GWAS SNP. Wild-type, glucose-raising alleles are circled in blue and the mutant, glucose-lowering alleles are circled in red. Diameter of the circle is proportional to the effect size estimates. Haplotype association was performed with FG derived residuals (after adjustment for age, sex, and BMI) using the most frequent haplotype as baseline.

The phenotypic impact of these coding variants might, we reasoned, be influenced by the action of the non-coding GWAS variants on *G6PC2* transcript regulation. Haplotype analysis of the four variants (rs560887, p.His177Tyr, p.Tyr207Ser, and p.Val219Leu) in a subset of 4,442 individuals revealed that the C allele encoding Leu219 was carried exclusively in *cis* with the glucose-raising allele at the GWAS SNP (Fig. 1). The estimated effect size of p.Val219Leu was considerably smaller ($\hat{\beta }$ =0.020 ± 0.004 mmol/L per allele) than rs560887 ($\hat{\beta }$ =0.070 ± 0.004 mmol/L per allele). These observations together explain the reversal in direction of effect of the Leu219 allele between conditional and unconditional analyses and indicate that, whilst Leu219 appears to have a glucose-raising effect in single-variant analyses, the molecular consequence of Leu219 is likely to be to lower glucose (Table 1). The minor alleles of the other two coding variants (p.His177Tyr, p.Tyr207Ser) also displayed glucose-lowering effects in conditional analyses. Given the role of G6PC2 in beta-cells we predicted that these would also be associated with reduced G6PC2 function, a hypothesis that we set out to test with a series of *in vitro* studies.

**Figure 2:**
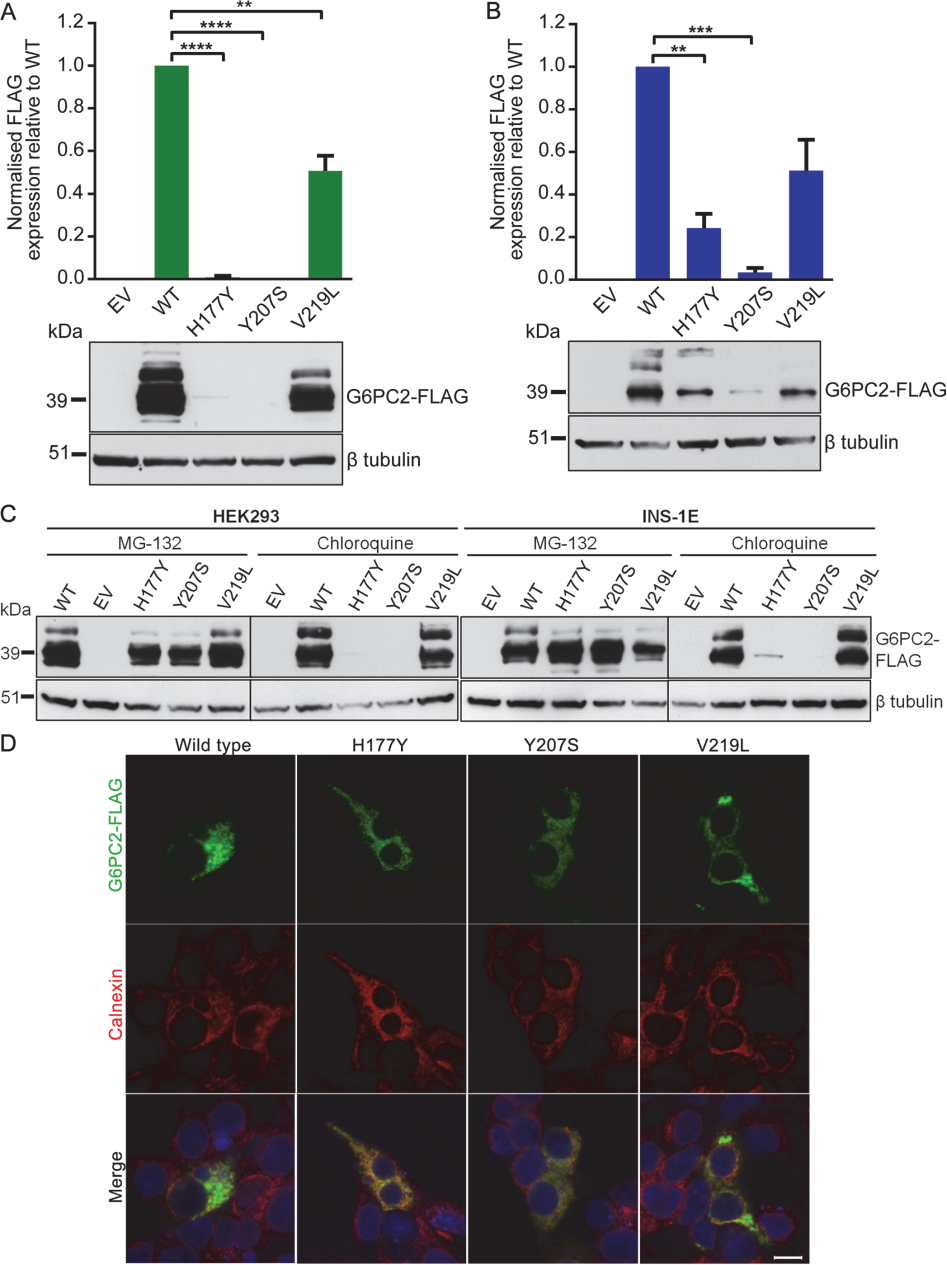
Functional characterization of wild type and variant G6PC2 proteins. (A) Expression levels in HEK293 and (B) INS-1E cells were determined by western blot and densitometry analysis. The multiple bands on the western blot are likely to represent glycosylated G6PC2 protein products. Data are presented as mean ± standard error of the mean for at least three independent experiments. Significant differences are indicated as ** *P*<0.01; *** *P*<0.001; **** *P*<0.0001. EV, empty vector; WT, wild type. (C) Expression levels in HEK293 and INS-1E cells in the presence of proteasomal inhibitor MG-132 or lysosomal inhibitor chloroquine were determined by western blot. (D) Cellular localization in HEK293 cells was assessed by immunofluorescence microscopy. Cells were double immunostained for FLAG tag (green) and calnexin (red), and merged images with a DNA stain (blue) are shown. Images were taken with laser settings that were optimized separately for each sample. Scale bar, 10µm.

First, we generated recombinant FLAG-tagged *G6PC2* constructs to investigate the impact of these variants on protein expression and subsequent cellular localization. Transient transfection of all three constructs showed a marked reduction in G6PC2 protein levels. In HEK293 cells, protein levels were decreased by 99% (p.His177Tyr), 100% (p.Tyr207Ser), and 49% (p.Val219Leu), when compared to wild type (defined for this study as the major allele for each of these variants) G6PC2 (Fig. 2A). Reduced protein expression was also confirmed in the rat pancreatic β-cell line INS-1E (76%, 97%, and 49% reduction, respectively; Fig. 2B). The functional impact of the variant proteins mirrored our genetic observations; the variant protein with the p.Val219Leu substitution, which has only a modest effect on FG, was present at higher levels in both HEK293 and INS-1E cells than the p.His177Tyr and p.Tyr207Ser proteins, both of which have larger impacts on FG levels.

Our functional results for p.His177Tyr and p.Tyr207Ser are concordant with data from the *in silico* mutation assessment tools SIFT and PolyPhen-2, which predicted these variants as deleterious (S4 Table). In contrast, the common p.Val219Leu variant was predicted to be benign, whereas *in vitro* characterization clearly shows a significant reduction in protein expression, demonstrating the importance of experimentally evaluating coding variants for functional consequences. The observed decrease in G6PC2 protein levels caused by the FG-associated variants is also directionally consistent with the evidence indicating that the non-coding GWAS SNP rs560887 may have effects on pre-mRNA splicing [19].

Next, we used specific inhibitors of the proteasomal and lysosomal pathways (MG-132 and chloroquine, respectively) to demonstrate that the three G6PC2 variant proteins with p.His177Tyr, p.Tyr207Ser, and p.Val219Leu substitutions were predominantly degraded through the ubiquitin-proteasome pathway, an important cellular mechanism for clearing misfolded proteins. Protein expression could be rescued in the presence of MG-132 but not chloroquine (Fig. 2C). We also evaluated the impact of the variants on cellular localization of G6PC2 to the endoplasmic reticulum (ER) using calnexin as an ER marker. The variant proteins displayed similar localization patterns to wild type G6PC2 (Fig. 2D). Our finding that loss of G6PC2 function leads to a reduction in FG levels in humans is consistent with rodent data, which show that *G6pc2* knockout mice have a ~15% decrease in FG levels [20]. Hence, our data, linking genetic associations with reduced protein function, indicate that normal G6PC2 function is critical for glucose homeostasis and that these variants most likely impact FG levels through altered intracellular catalysis of glucose-6-phosphate to glucose and inorganic phosphate in pancreatic β-cells.

### Impact of the functional *G6PC2* variants on other related quantitative traits and disease outcomes

We evaluated the impact of these functional *G6PC2* variants on other related quantitative traits and disease outcomes to gain further insights into the metabolic processes involved (S7 Table). None of the variants showed any evidence of association with FI levels (*P*>0.1) in 30,825 non-diabetic individuals analyzed in the present study. No association of the lead *G6PC2* GWAS variant with T2D risk has previously been shown in Europeans [2, 21, 22]. However, in a meta-analysis of exome-array data from 28,344 T2D cases and 51,801 controls of European ancestry (including the 33,231 controls used in the present meta-analyses), the common coding variant, p.Val219Leu, showed modest association with T2D risk (*P* = 0.0011; odds ratio = 1.05, 95% confidence interval 1.02–1.06). The G allele encoding Val219, which displayed a glucose-raising effect, conferred an increased risk of disease. In contrast to the observations in FG single-variant analysis, the direction of effect on T2D at this variant was unchanged, even after conditioning on the GWAS variant. This is consistent with a small case-control study in 3,676 individuals of Chinese ancestry where a nominal association (P = 0.0062) with T2D risk was reported [23]. Our finding provides evidence that *G6PC2* is critical not only for controlling FG levels in the physiological range, but that impairment of its function contributes to T2D pathogenesis. The effect on T2D risk is modest given the impact of the p.Val219Leu variant on FG levels but the effect size is similar to that reported for the common non-coding variant GWAS signal at *GCK* [2]. *GCK* encodes the glycolytic enzyme glucokinase which catalyzes the reverse reaction to G6PC2. These observations are consistent with defects in glucose sensing rather than β-cell function.

### Likely effector transcripts at established FG GWAS loci

Of the 12 coding variants significantly influencing FG levels, we have discussed eight above. The four remaining signals mapped to three additional established FG-associated GWAS regions and support the candidacy of particular effector transcripts at these loci. Two map to *PCSK1* (p.Gln665Glu, MAF = 27.9%, *P* = 3.0×10^−8^; p.Ser690Thr, MAF = 27.9%, *P* = 4.1×10^−8^), and have previously been proposed as likely causal variants at this GWAS locus because of strong LD with the lead non-coding GWAS SNP (rs4869272) in Europeans (*r^2^* = 0.81) [3]. These two variants are in complete LD with each other (*r^2^* = 1) and both have residual association signals after adjusting for the lead GWAS SNP (p.Gln665Glu, *P*_conditional_ = 8.1×10^−3^; p.Ser690Thr, *P*_conditional_ = 0.01) (Table 1 and S3 Table). Conversely, conditioning on the coding variants completely abolished the association signal at the GWAS SNP (*P_unconditional_* = 7.2×10^−7^; *P_conditional on_*_Gln665Glu_ = 0.24, *P_conditional on_*_Ser690Thr_ = 0.25, and *P_conditional on_*_Gln665Glu and Ser690Thr_ = 0.34). Although we were unable to statistically distinguish between these two coding variants, there is suggestive *in vitro* evidence that the p.Gln665Glu variant may decrease PCSK1 activity whilst p.Ser690Thr behaved as wild-type [24], supporting the former as the most likely causal variant at the *PCSK1* locus. *PCSK1* encodes prohormone convertase 1/3 (PC1/3), a calcium-dependent serine endoprotease, which is essential for the conversion of a variety of prohormones, including proinsulin and proglucagon, to their bioactive forms.

The penultimate variant was in *ZHX3* (p.Asn310Ser, MAF = 23.8%, *P* = 3.9×10^−7^) which maps to the *TOP1* GWAS locus influencing FG levels [4]. Adjustment for p.Asn310Ser in a conditional analysis eliminated the association signal at the non-coding GWAS lead SNP rs6072275 (*P*_unconditional_ = 2.5×10^−5^ and *P*_conditional_ = 0.33), supporting *ZHX3* as the plausible causal gene at this locus (Table 1 and S3 Table). *ZHX3* (zinc fingers and homeoboxes 3) encodes a transcriptional repressor which belongs to a protein family known to regulate gene expression in the kidney podocytes and plays roles in both lipoprotein metabolism and triglyceride regulation in mice [25, 26].

The final variant was in *RREB1* (p.Ser1554Tyr, MAF = 21.1%, *P* = 8.4×10^−9^) which resides in the *RREB1*/*SSR1* GWAS locus influencing FG levels [4] and T2D risk [27] (Table 1). We were unable to explore the relationship between this association signal and the lead FG (rs17762454) or T2D (rs9502570) GWAS SNPs at this locus as neither the variants themselves, nor a close proxy (*r^2^*>0.80), was present on the exome-array. Based on European haplotypes from the 1000 Genomes Project, the coding variant was more strongly correlated (*r^2^* = 0.59) with rs9502570 as compared to rs17762454 (*r^2^* = 0.08), which might indicate multiple FG association signals in this region. These data point to a likely functional role for *RREB1* at this locus, although further studies are needed to confirm this hypothesis.

### Coding variants influencing FI levels

Turning to the analysis of FI, we identified six non-synonymous variants associated at exome-wide significance (one rare and five common). These mapped to four loci, three of them previously implicated in FI regulation (S3 Table). The single novel FI- influencing locus was represented by a rare variant in *URB2* (p.Glu594Val, MAF = 0.1%, *P* = 3.1×10^−7^; Table 1 and S1 Fig.). The variant allele was observed in individuals across Europe, including UK, Denmark, Finland, and Sweden, and genotype calls across all cohorts passed visual inspection for clustering accuracy. Heterozygous genotypes (n = 6) were 100% concordant for 3,999 individuals genotyped on the array that had also been exome sequenced (S8 Table). This variant has a relatively large effect, with each copy of the minor allele increasing FI level by 32%. *URB2* encodes ribosome biogenesis 2 homolog, which interacts with nuclear lamins [28]. The precise mechanistic role of *URB2* is still poorly understood, but conditions caused by defects in lamin genes (laminopathies), including familial partial lipodystrophy, can cause loss of adipose tissue, insulin resistance, and metabolic syndrome [29, 30].

The remaining five coding variants implicated in the FI analysis, included three previously-reported FI-associated common variants in *GCKR* (p.Pro446Leu, *P* = 8.1×10^−11^), *PPARG* (p.Pro12Ala, MAF = 14.7%, *P* = 1.3×10^−7^), and *COBLL1* (p.Asn939Asp, MAF = 11.3%, *P* = 6.7×10^−8^) [3, 4]. The final two variants (in *C2orf16* and *GPN1*) also mapped within the *GCKR* FG/FI-associated GWAS locus, and their associations were eliminated after conditioning on the *GCKR* p.Pro446Leu variant as seen in FG analysis (S3 Table).

## Discussion

In the current study, we have examined the contribution of coding variants in influencing glycemic trait levels and identified significant associations at ten loci, highlighting multiple functional genes.

We have established a functional role for *G6PC2* in FG homeostasis and have identified two novel loci associated with glycemic traits: *GLP1R* and *URB2*. In three additional GWAS regions for FG, we have identified potentially causal coding variants, highlighting likely effector transcripts (*PCSK1*, *RREB1*, and *ZHX3*). In line with earlier observations, very few of the FG- and FI-associated loci identified impact T2D risk [4]. The exception in this study is the p.Val219Leu *G6PC2* variant, which showed a modest but directionally consistent association with T2D risk, in contrast to earlier reports suggesting that, paradoxically, glucose-raising alleles at this GWAS locus are protective against T2D [2].

Our results have a number of important implications for the design, analysis, and interpretation of association studies of coding variation. First, we have demonstrated that an appreciation of regional haplotypes is fundamental to understanding the relationship between genetic variation and gene function, phenotype, and disease. In this study, haplotype analysis was essential in resolving the apparent discrepancy between the observed phenotypic effect driven by the *G6PC2* C allele at the variant encoding Leu219 and our *in vitro* data. From a clinical genomics perspective, this example illustrates how interpretation of single-variant analyses can be misleading and that the explicit phase relationships of variants are important for the correct interpretation of allelic effects. Furthermore, they highlight the importance of considering the possible interactions between regulatory and coding variants on protein levels [31]. The haplotype analysis also allows us to define specific regional diploid haplotypes that differ markedly in glucose levels. This is particularly relevant to efforts to follow up these variants in human data, whether for *in vivo* physiology, or for cellular studies on biopsies or patient derived stem cells.

Second, we have highlighted some limitations of widely used *in silico* prediction programs in accurately evaluating the impact of genomic variation on protein function. Earlier studies have shown that filtering functional variants based on *in vitro* data can substantially improve the power to detect causal genes [32, 33]. When such data are not available, *in silico* predictions can be used to help identify variants of functional significance [10, 34]. However, as we have demonstrated with the *G6PC2* p.Val219Leu variant, current *in silico* predictions are not always accurate and should be validated with *in vitro* functional analysis wherever possible.

We find little support for a widespread impact of rare or low-frequency coding variants in accounting for known common SNP FG- and FI-associated GWAS signals. However, given that exome-array genotyping does not capture the complete repertoire of coding variation throughout the genome, exome sequencing is still required to achieve a complete assessment of their impact on glycemic traits at all associated loci. It is also likely that analysis of non-coding regulatory variation will be necessary to elucidate the causal genes and functional roles of association signals observed in prior GWAS. However, our study provides clear examples of how the analysis of coding variation can aid interpretation of GWAS findings where biology was previously unclear, and highlights the promise of this approach to provide insights into the pathophysiology of common complex disease.

### Data availability

Summary statistics of single-variant and gene-based analyses are available at http://www.diagram-consortium.org/Mahajan_2014_ExomeChip/

## Materials and Methods

### Ethics statement

All human research was approved by the relevant institutional review boards, and conducted according to the Declaration of Helsinki and all patients provided written informed consent. FIN-D2D 2007, DPS, DR’s EXTRA, FINRISK 2007, FUSION, and METSIM were approved by the University of Michigan Health Sciences and Behavioral Sciences Institutional Review Board (ID: H03-00001613-R2). The Danish studies (Health 2006, Inter99, and Vejle Biobank) were approved by the local Ethical Committees of Capital Region (approval # H-3-2012-155, KA 98155 and KA-20060011) and Region of Southern Denmark (approval # S-20080097). The GoDARTS study was approved by EoS REC 09/S1402/44. The Twins UK study was approved by EC04/015. The OBB study was a approved by South Central, Oxford C, 08/H0606/107+5, IRAS project 136602. The PIVUS study is approved by 00–419 and ULSAM study by 251/90 and 2007/338. The PPP study was approved by the Committee On the Use of Humans as Experimental Subjects at MIT (IRB 0912003615).

### Phenotypes

**Study participants.** FG was measured in mmol/L and FI in pmol/L. Individuals were excluded from the analysis if they had a physician diagnosis of diabetes, were on diabetes treatment (oral or insulin), or had a fasting plasma glucose concentration ≥7 mmol/L or ≥11.1 mmol/L following a 2-hour oral glucose tolerance test. Individual studies applied further sample exclusions, including for pregnancy, non­fasting measurements, and type 1 diabetes (S1 Table). Measures of fasting glucose made in whole blood were corrected to approximate plasma level by multiplying by 1.13 [35].

**Trait transformations and adjustment.** To achieve approximate normality of the traits within each study, FG and natural logarithm–transformed FI levels were adjusted for age, sex, BMI, and study specific covariates followed by inverse normalization of the residuals. Inverse normalized residuals were used as the dependent quantitative trait in genetic association models to calibrate type 1 error. Effect estimates were obtained using untransformed FG and natural logarithm–transformed FI levels after adjusting for age, sex, BMI, and study specific covariates.

### Genotyping and quality control

The Illumina HumanExome Beadchip array was genotyped by individual studies. This custom array was designed to facilitate large-scale genotyping of 247,870 mostly rare (MAF<0.5%) and low-frequency (MAF 0.5–5%) protein altering variants selected from sequenced exomes and genomes of ~12,000 individuals (see URLs). Details of genotype calling and quality control are presented in S1 Table. To confirm the genotyping quality of all the variants discussed in the manuscript, we compared the genotype calls in 2,312 to 4,000 individuals for which we have both exome-array genotypes and exome-sequence data (S8 Table). The results are highly concordant for all variants (99% heterozygous genotype concordance and 100% concordance observed for non-reference homozygotes). Call rate and Hardy-Weinberg equilibrium p-values for each variant are provided in S9 Table.

### Association analysis

**Single-variant analysis.** We tested single variants for association with FG- and FI-derived inverse normalized residuals assuming an additive genetic model using a linear mixed model to account for relatedness with EMMAX [5]. Study-specific QQ plots and genomic lambdas are shown in the S3 Fig. We repeated single-variant association analyses with untransformed FG and natural logarithm–transformed FI residuals to obtain effect estimates. We then combined the association summary statistics across studies by using a fixed-effects meta-analysis (sample size Z-score weighted) using METAL [36]. Genotype cluster plots of all variants described here were inspected visually in all studies.

**Single-variant conditional analysis.** Conditional analysis was performed to identify additional association signals at known or novel loci. The analysis included the genotypes of the lead variant(s) at the conditioning loci as covariate(s) in the regression analysis in EMMAX. We then performed meta-analysis of the association summary statistics across studies by using a fixed-effects meta-analysis (sample size Z-score weighted).

**Gene-based analysis.** For gene-based testing, we first computed single-variant score statistics and their covariance matrices for all polymorphic markers within each study. We then combined the single-variant score statistics from all studies using the Cochran-Mantel-Haenszel method and computed corresponding variance-covariance matrices centrally [6]. These variance-covariance matrices were used to compute gene-level statistics. We applied a frequency-weighted burden test which assumes variants have similar effect sizes and SKAT, a dispersion test that performs well when both protective and deleterious variants are present [8, 9]. Test-specific asymptotic distributions were used to evaluate significance. For gene-based analyses, we used only unrelated individuals (n = 32,223 for FG and n = 29,848 for FI) and included principal components as covariates to adjust for population structure. We generated four variant lists using frequency data and functional annotation. Variants were mapped to transcripts in Ensembl 66 (GRCh37.66). Using annotations from CHAoS v0.6.3, SnpEff v3.1, and VEP v2.7, we identified variants predicted to be protein-truncating (PTV; e.g. nonsense, frameshift, essential splice site) or protein-altering (e.g. missense, in-frame indel, non-essential splice site) in at least one mapped transcript (by at least one of the three algorithms) [37, 38]. We additionally used the procedure described by Purcell *et al*. to identify subsets of missense variants meeting “strict” or “broad” criteria for being deleterious, using annotation predictions from Polyphen2-HumDiv, PolyPhen2-HumVar, LRT, MutationTaster, and SIFT [10]. Masks 1 (“PTV-only”) and 3 (“PTV + NS_strict_”) are restricted to variants with predicted major effect on protein function, and, as a result, disproportionately favor inclusion of rare variants, whilst masks 2 (“PTV + missense”) and 4 (“PTV + NS_broad_”) are more permissive. In total, we tested 1,028, 14,465, 4,603, and 13,093 genes having at least two such variants in the above four categories respectively. For gene-based tests reaching exome-wide significance, if the conditional analysis showed that the signal was driven by a single variant, we required the variant to achieve exome-wide significance in the single-variant test as well.

**Gene-based conditional analysis.** We performed conditional gene-level analysis to evaluate whether rare or low-frequency variants associated with the trait in single-variant analysis could account for or were due to a gene-based test association signal [6]. The genotypes of the variant(s) at the conditioning locus were included as covariate(s) in this analysis.

**Estimating phenotypic variance explained by SNPs.** We used a subset of Finnish samples (FIN-D2D 2007, DPS, DR’s EXTRA, FINRISK 2007, and METSIM; n = 10,266) to calculate variance explained by *G6PC2*. We ran a model regressing BMI adjusted FG on the four *G6PC2* variants: intronic GWAS lead SNP rs560887, p.His177Tyr (rs138726309), p.Tyr207Ser (rs2232323), and p.Val219Leu (rs492594), assuming an additive model (and adjusting for sex, age, age^2^, and study origin). A separate model was run excluding the GWAS SNP to determine the additional variance captured with the three coding variants.

**Power calculations.**We had >99.9% power to identify variants that explain >0.3% of the phenotypic variance and 80% power to detect coding variants that explain >0.1% of the phenotypic variance. To achieve >80% power for variants with MAF<0.05% would require effect sizes of at least 1 SD unit of residuals of mmol/L for FG and pmol/L for FI.

When we estimate power to detect association for aggregation tests, we make many assumptions: i) proportion of variants contributing to trait variation, ii) direction of effects, iii) number of variants aggregated, and iv) allele frequency distribution of the variants [34]. In this study, assuming 100% of the variants contribute to trait variation, we had >99.9% power to detect association for genes that explain >0.25% of the phenotypic variance and 80% to detect genes that explain >0.1% of the phenotypic variance using a burden test and >0.5% of the phenotypic variance using SKAT. In a less favorable scenario, for example assuming a gene explains 0.25% of the phenotypic variance, 25% of the variants contribute to trait variation, different directions of effect, 20 variants tested, and variants sampled from the reported allele frequency distribution in the exome-array design, power to detect association in this study may be near 0% for a burden test and 63% for SKAT [34].

**Haplotype analysis.** In 4,442 individuals from the Oxford Biobank, we used an expectation-maximization (EM) algorithm to obtain the posterior distribution of haplotypes consistent with the observed genotypes at four *G6PC2* variants: intronic GWAS lead SNP rs560887, p.His177Tyr (rs138726309), p.Tyr207Ser (rs2232323), and p.Val219Leu (rs492594). Haplotype association with FG- and FI-derived residuals (after adjustment for age, sex, and BMI) was tested in a linear regression framework, as a function of haplotype dosage from posterior distribution, and including principal components as covariates to account for population structure using the most frequent haplotype as baseline.

***In-silico* mutation analysis.** SIFT [39], PolyPhen-2 [40], and Condel [41] algorithms were used to predict the functional effects of the associated non-synonymous variants on protein function. Genomic Evolutionary Rate Profiling (GERP) [42] scores were calculated to indicate the degree of evolutionary conservation at a given human nucleotide based on multiple genomic sequence alignments and were measured as site-specific ‘rejected substitutions’: higher scores indicate greater conservation.

### Functional studies

**Site directed mutagenesis.** Human *G6PC2* cDNA (NM_021176.2) within a pCMV6-Entry vector (with a C-terminal FLAG-tag) was purchased from OriGene (RC211146). We generated non-synonymous variants in the *G6PC2* coding sequence of the clone using Quikchange II Site-Directed Mutagenesis (Agilent). All mutations were verified by Sanger sequencing; in each case, only the desired nucleotide substitutions was introduced.

**Western blot analyses.** HEK293 and INS-1E 832–13 cells were transfected with each FLAG-tagged wild type or mutant G6PC2 construct using Lipofectamine 2000 (Invitrogen). In protein degradation assays, cells were treated with 10μM MG-132 (Calchembio) or 100μM chloroquine (Sigma) for 15h. At 48h after transfection, cells were collected and homogenized by sonication in lysis buffer. Total cellular protein was separated by 4–12% SDS-PAGE (Invitrogen) and transferred to nitrocellulose membranes. We determined G6PC2 expression by immunoblotting using a mouse monoclonal FLAG M2 antibody (Sigma, F1804). A rabbit polyclonal antibody against β tubulin (Santa Cruz, sc-9104) was used as a loading control. Secondary antibodies specific to mouse or rabbit IgG were obtained from Thermo Fisher Scientific. Protein bands were detected using the ECL reagent (Pierce Thermo Fisher Scientific). Western blots were quantified by densitometry analysis using ImageJ and paired t tests of densitometric data were carried out in GraphPad Prism.

**Immunofluorescence.** HEK293 cells were transfected with each FLAG-tagged G6PC2 construct using FuGene 6 transfection reagent (Promega) in 4-well chamber slides (BD Biosciences). After 48h, cells were fixed with 4% paraformaldehyde in PBS for 15 min. Cells were permeabilized with 0.05% Triton X-100 in PBS for 15 min, and blocked for 1 h with 10% BSA in PBS-Tween 20. We carried out double immunostaining of cells using FLAG M2 (Sigma, F1804) and calnexin (Santa Cruz, sc-11397) primary antibodies followed by anti-mouse fluorescein (Vector Labs) and anti-rabbit TRITC (Dako). DRAQ5 fluorescent probe (Thermo Fisher Scientific) was applied at 20μM as a far-red DNA stain. Slides were mounted with Vectashield mounting medium (Vector Labs) and visualized on a BioRad Radiance 2100 confocal microscope with a 60× 1.0 N.A. objective. Images were acquired with different laser settings that were optimized for each sample and therefore fluorescent intensities are not comparable across samples.

### URLs

Exome-array design,
http://genome.sph.umich.edu/wiki/Exome_Chip_Design
EPACTS,
http://genome.sph.umich.edu/wiki/EPACTS
METAL,
http://www.sph.umich.edu/csg/abecasis/Metal/download/
RareMETALS,
http://genome.sph.umich.edu/wiki/RareMETALS
The 1000 Genomes Project,
www.1000genomes.org

## Supporting Information

S1 Fig Forest plots of association results with fasting glucose (FI) and fasting insulin (FG) levels. Individual study and meta-analysis effects of A) *URB2* coding variant rs141203811 (p.Glu594Val) on FI; B) *GLP1R* coding variant rs10305492 (p.Ala316Thr) on FG; and C), D), & E) *G6PC2* coding variants rs138726309 (p.His177Tyr), rs2232323 (p.Tyr207Ser), rs492594 (p.Val219Leu) on FG.
(PDF)

S2 Fig Regional association plots for FG at *G6PC2* region on chromosome 2. (A) Unconditional association results highlight the previously known non-coding lead SNP rs560887. (B) Association results after conditioning on rs560887 highlight two non-synonymous coding variants rs138726309 (p.His177Tyr) and rs492594 (p.Val219Leu), both largely independent from the signal from rs560887. (C) Association results conditioning on rs560887 (GWAS SNP) and rs138726309 (p.His177Tyr) highlights rs492594 (p.Val219Leu) as an independently associated variant at *G6PC2*. (D) Association results conditioning on rs560887 (GWAS SNP) and rs492594 (p.Val219Leu) highlights rs138726309 (p.His177Tyr) as the second independently associated coding variant at *G6PC2*
(PDF)

S3 Fig Quantile-quantile plots (qq-plot) for each study included in the meta-analysis for FG and FI. Genomic control (GC) is given in each qq-plot.
(PDF)

S1 Table Characteristics of the study cohorts. (DOCX)

S2 Table Sample characteristics of the study cohorts. (DOCX)

S3 TableCoding variants achieving exome-wide significant association with FG and FI levels in single-variant analysis.MAF: minor allele frequency. $\hat{\beta }$: regression coefficient estimates. SE: standard error. N: number of samples analyzed. I^2^: heterogeneity measure in %. *P*_het: P-value for Cochran’s Q statistic. ^a^Sample-size weighted average minor allele frequency percentage across all studies.(DOCX)

S4 Table *In silico* predictions of the impact of the non-synonymous changes on protein function and the evolutionary conservation of variants. GERP: Genomic Evolutionary Rate Profiling
(DOCX)

S5 Table Conditional analysis of genes associated with FG and FI levels at exome-wide significance in gene-based tests using SKAT and BURDEN. * Although *AKT2* reached exome-wide significance, the signal was driven by a single variant which showed only suggestive significance in single-variant analysis (*P*=9.3×10^−7^). As described in the methods, we required the single-variant test to be exome-wide significant (*P*<5×10^−7^) in such a scenario
(DOCX)

S6 Table FG single-variant association results of the rare and low-frequency G6PC2 coding variants included in the gene-based tests based on unrelated samples. MAF: minor allele frequency; Allele counts: Minor allele counts. ^a^Alleles are aligned to the forward strand of NCBI Build 37. ^b^Sample-size weighted average minor allele frequency percentage across all studies. *P* values are obtained with derived inverse normalized residuals of mmol/L of fasting glucose after adjustment for age, sex, and BMI. Direction of effect is aligned to the minor allele.
(DOCX)

S7 Table Association with T2D, birth weight and diabetes-related quantitative traits. m: Minor allele; M: Major allele; BMI: Body mass index; WHR: Waist-hip ratio; FG: Fasting glucose level; FI: Fasting insulin level, adjusted for BMI; HbA1c: Hemoglobin A1-C level; HOMA-B: Homeostasis model assessment-B score; HOMA-IR: Homeostasis model assessment-insulin resistance; SBP: Systolic blood pressure; DBP: Diastolic blood pressure; TG: Triglycerides; HDL-C: HDL cholesterol; LDL-C: LDL cholesterol; TC: Total cholesterol; BW: Birth weight. (a) Trait increasing allele in bold. (b) Samples contributing to T2D case-control analysis include a subset of the non-diabetic samples contributing to the current analysis. (c) All published data is “unconditioned”. As seen in our analysis, direction of effect switches after conditioning on rs560887 at least for FG. For exome-chip FG and T2D we have provided results from conditional analysis and G is the trait increasing allele.
* rs2232323 (*G6PC2*), rs138726309 (*G6PC2*), rs35742417 (*RREB1*), and rs141203811 (*URB2*) have not been investigated in earlier GWAS
(DOCX)

S8 Table Genotype concordance of all the variants discussed in the manuscript. Concordance is computed for the variants with non-missing calls in both exome-array and exome sequence data. N: Number of samples.
(DOCX)

S9 Table Study-wise call rate and Hardy-Weinberg equilibrium P-values of all the variants discussed in the manuscript. CR: call rate. HWE: Hardy-Weinberg equilibrium p-value.
(DOCX)
