# Supplementary figures and images for: Identification and Functional Characterization of G6PC2 Coding Variants Influencing Glycemic Traits Define an Effector Transcript at the G6PC2-ABCB11 Locus
Source: PLoS Genet. 2015 Jan 27;11(1):e1004876. doi: 10.1371/journal.pgen.1004876 (PMC4307976; doi:10.1371/journal.pgen.1004876)

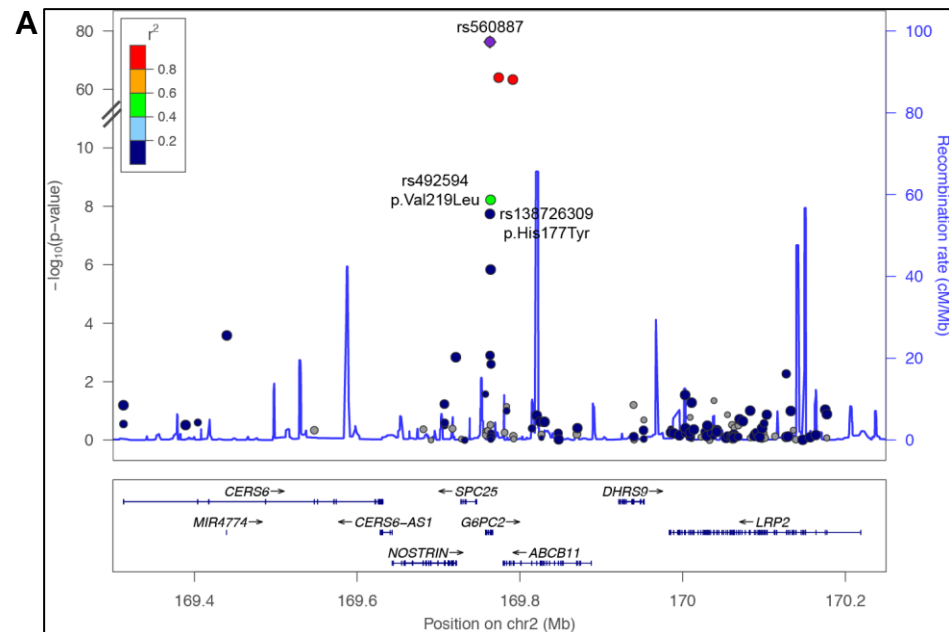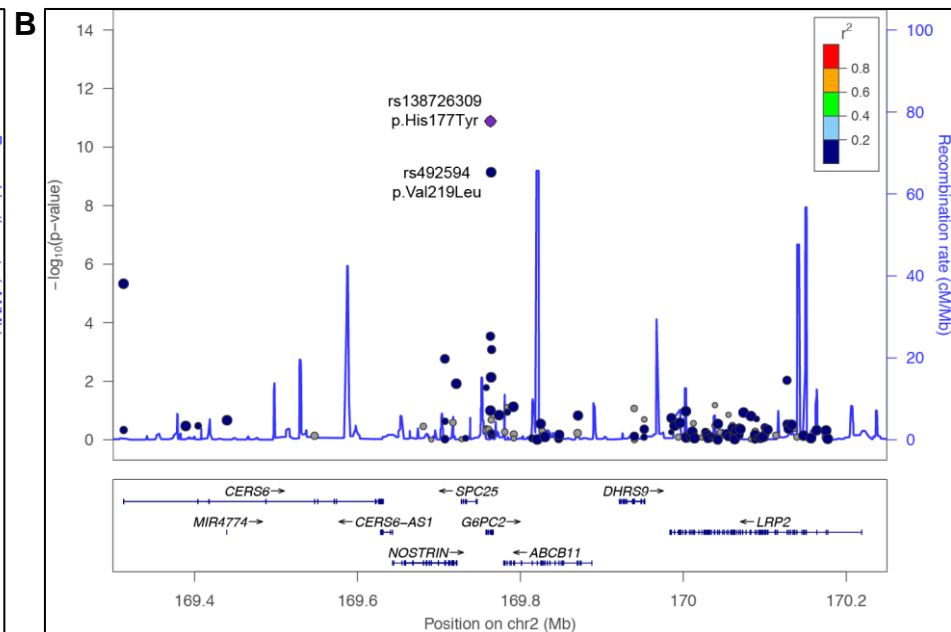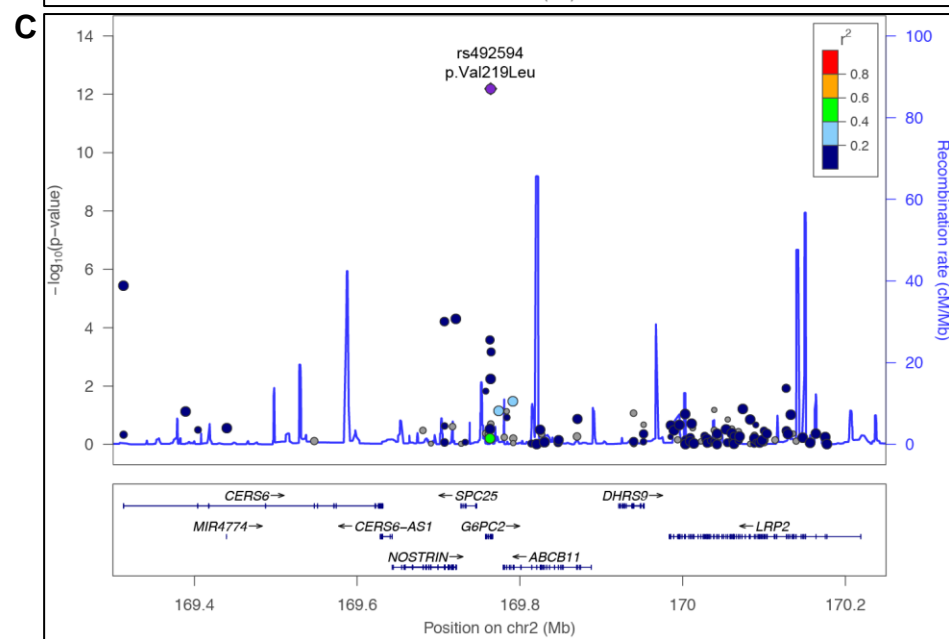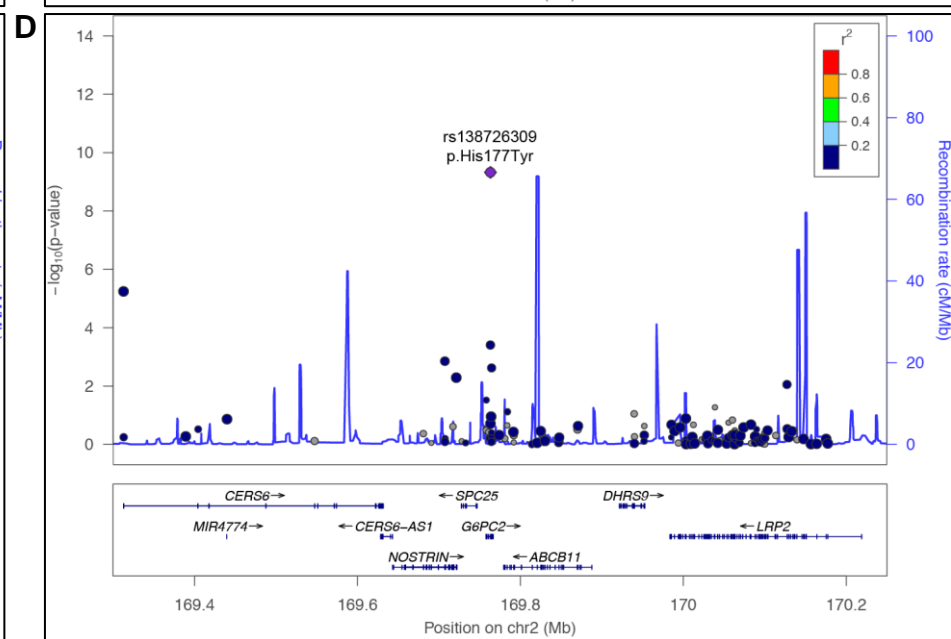

Supplement: S1 Fig — Individual study and meta-analysis effects of A) URB2 coding variant rs141203811 (p.Glu594Val) on FI; B) GLP1R coding variant rs10305492 (p.Ala316Thr) on FG; and C), D), & E) G6PC2 coding variants rs138726309 (p.His177Tyr), rs2232323 (p.Tyr207Ser), rs492594 (p.Val219Leu) on FG. (PDF) [file pgen.1004876.s001.pdf]

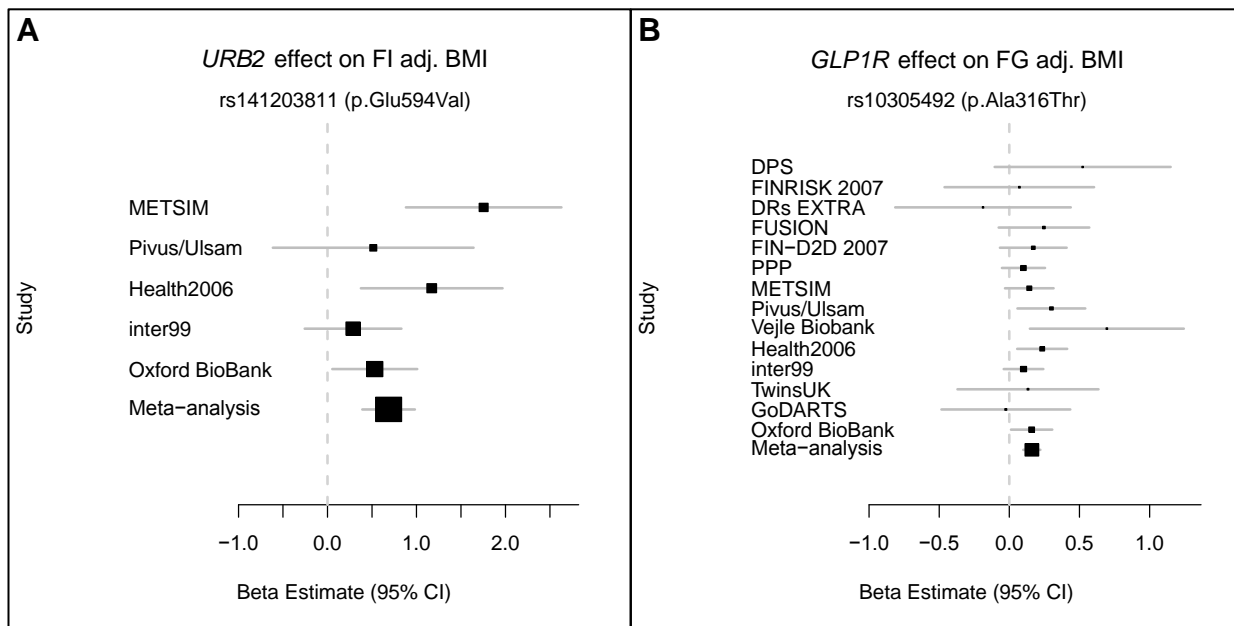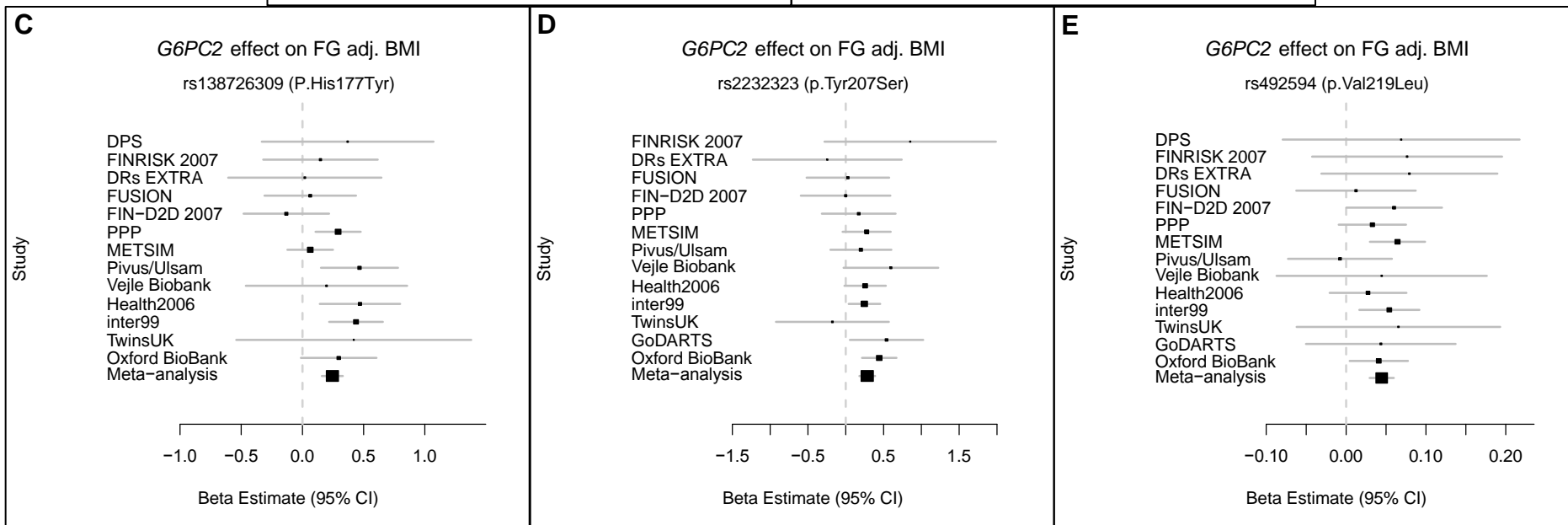

Supplement: S2 Fig — (A) Unconditional association results highlight the previously known non-coding lead SNP rs560887. (B) Association results after conditioning on rs560887 highlight two non-synonymous coding variants rs138726309 (p.His177Tyr) and rs492594 (p.Val219Leu), both largely independent from the signal from rs560887. (C) Association results conditioning on rs560887 (GWAS SNP) and rs138726309 (p.His177Tyr) highlights rs492594 (p.Val219Leu) as an independently associated variant at G6PC2. (D) Association results conditioning on rs560887 (GWAS SNP) and rs492594 (p.Val219Leu) highlights rs138726309 (p.His177Tyr) as the second independently associated coding variant at G6PC2 (PDF) [file pgen.1004876.s002.pdf]

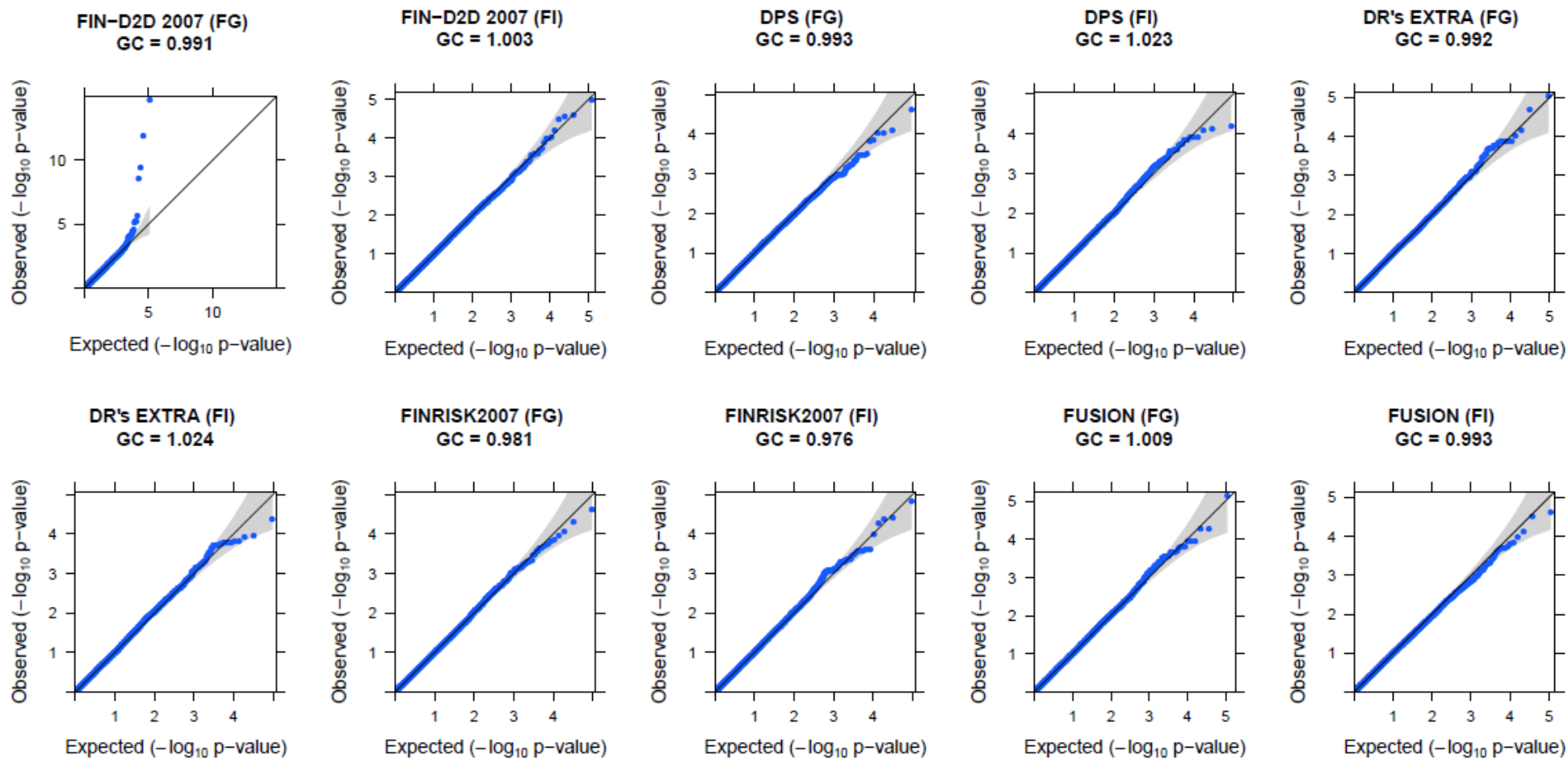

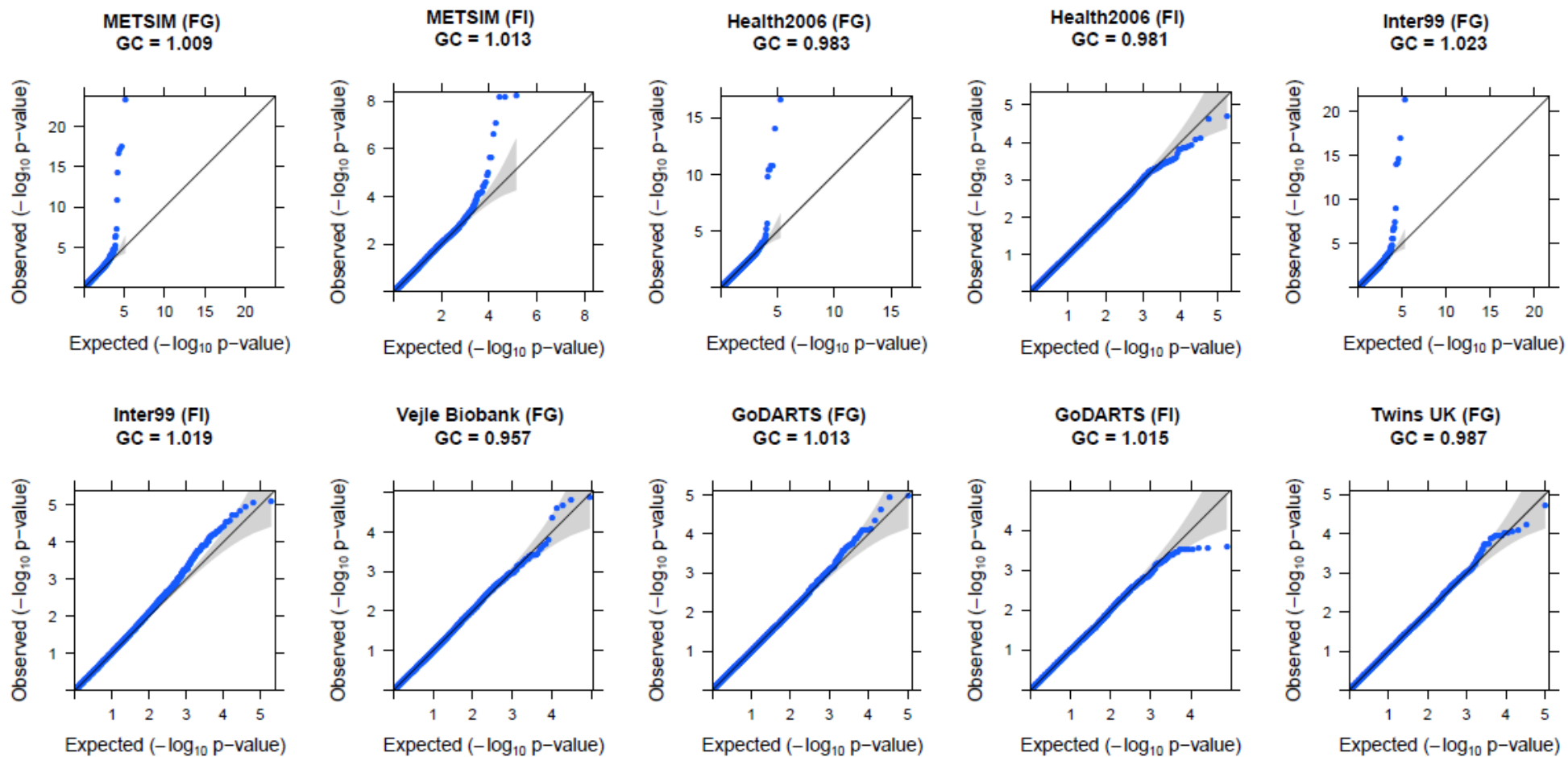

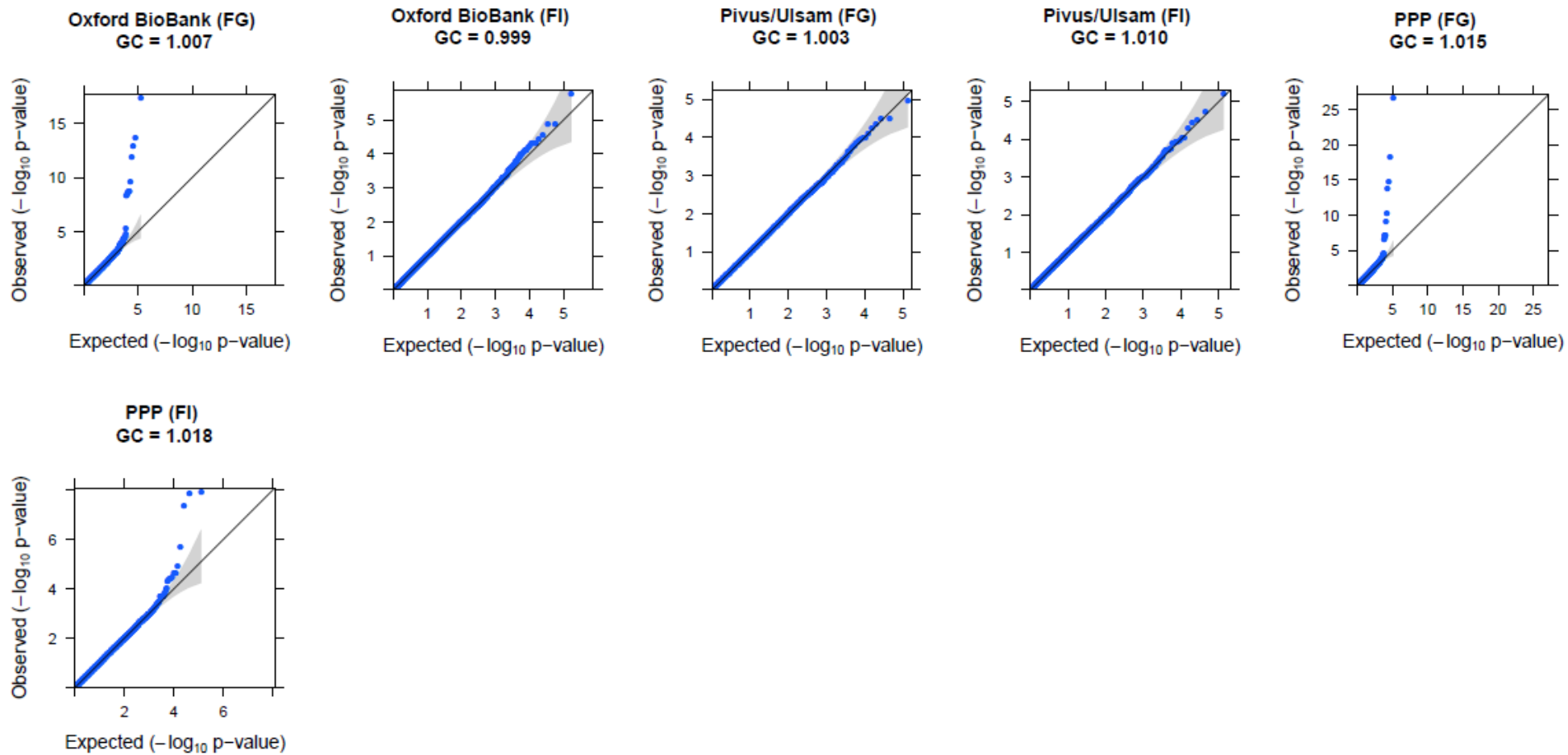

Supplement: S3 Fig — Genomic control (GC) is given in each qq-plot. (PDF) [file pgen.1004876.s003.pdf]
