# Supplementary material for: Identification and Functional Characterization of G6PC2 Coding Variants Influencing Glycemic Traits Define an Effector Transcript at the G6PC2-ABCB11 Locus
Source: PLoS Genet. 2015 Jan 27;11(1):e1004876. doi: 10.1371/journal.pgen.1004876 (PMC4307976; doi:10.1371/journal.pgen.1004876)
Supplement: S1 Table — (DOCX) [file pgen.1004876.s004.docx]

|  |  |  |  |  | **Sample QC** | | **Variant QC** | | | |  |
| --- | --- | --- | --- | --- | --- | --- | --- | --- | --- | --- | --- |
| **Ancestry** | **Study** | **Citation (s)** | **Sample Ascertainment** | **Genotyping**  **array** | **Call rate** | **Exclusion criteria** | **Call rate** | **Filtering criteria** | **Calling algorithm** | **Number of variants analysed (N) for FG and FI**  **[% of variants in frequency bins:**  **MAF < 0.05% /**  **0.05% ≤ MAF < 5% / MAF ≥ 5%]** | **Association covariates** |
| **European [Finnish]** | FIN-D2D 2007 | 20459722 [43] | - Population-based survey - Glucose tolerance classified according to WHO 1999 criteria - T1D and T2D (fasting plasma glucose concentration ≥7.0 mmol/l or 2-h plasma glucose concentration ≥11.1 mmol/l) cases excluded - Further excluded individuals with HbA1c ≥6.5% according to ADA 2012 criteria for T2D | HumanExome-12v1-1_A | >99% | - call rate ≤99% - heterozygosity >median + 3*IQR - technical duplicates with lower call rate - Non-European population outliers - sex discrepancy - contamination score >10% | ≥95% | - exclude 101 indels with different allele mapping across the two sites - exclude variants that had chromosome or position or allele mismatch - exclude duplicated variants, keeping the one with higher call rate. If the call rate is the same, arbitrarily take the one with that comes first in the file - call rate <95% - exact HWE <10^-5^ | Illumina GenCall using standard Illumina cluster files + Zcall | FG  N = 59,745 [0.00/56.90/43.10]  FI  N = 59,647 [0.00/56.82/43.18] | - age, age2, sex, BMI for EMMAX-analysis - age, age2, sex, BMI, PC1, PC2, PC3, PC4 for rvtest analysis |
| **European [Finnish]** | The Finnish Diabetes Prevention Study (DPS) | 11333990 [44] | - Randomised controlled trial - All subjects were impaired glucose tolerant at baseline, from mean of two OGTTs using WHO 1985 criteria - Excluded individuals with fasting plasma glucose ≥7.0 mmol/l or 2-h plasma glucose concentration ≥11.1 mmol/l or HbA1c ≥6.5% according to ADA 2012 criteria for T2D | HumanExome-12v1_A | >99% | - call rate ≤99% - heterozygosity >median + 3*IQR - technical duplicates with lower call rate - Non-European population outliers - sex discrepancy - contamination score >10% | ≥95% | - exclude 101 indels with different allele mapping across the two sites - exclude variants that had chromosome or position or allele mismatch - exclude duplicated variants, keeping the one with higher call rate. If the call rate is the same, arbitrarily take the one with that comes first in the file - call rate <95% - exact HWE <10^-5^ | Genotype calls generated on cluster boundaries trained on using study samples + manual review of clusterplots | FG  N = 44,140 [0.00/40.04/59.96]  FI  N = 43574 (0.00/39.31/60.69) | - age, age2, sex, BMI for EMMAX-analysis - age, age2, sex, BMI, PC1, PC2, PC3, PC4 for rvtest analysis |
| **European [Finnish]** | The Dose Responses to Exercise Training (DR's EXTRA) Study | 21186108 [45] | - Randomised controlled trial - Glucose tolerance classified according to WHO 1999 criteria - T1D and T2D (fasting plasma glucose concentration ≥7.0 mmol/l or 2-h plasma glucose concentration ≥11.1 mmol/l or physician diagnosed) cases excluded | HumanExome-12v1-1_A | >99% | - call rate ≤99% - heterozygosity >median + 3*IQR - technical duplicates with lower call rate - Non-European population outliers - sex discrepancy - contamination score >10% | ≥95% | - exclude 101 indels with different allele mapping across the two sites - exclude variants that had chromosome or position or allele mismatch - exclude duplicated variants, keeping the one with higher call rate. If the call rate is the same, arbitrarily take the one with that comes first in the file - call rate <95% - exact HWE <10^-5^ | Illumina GenCall using standard Illumina cluster files + Zcall | FG  N = 46,656 (0.00/45.07/54.93)  FI  N = 47,062 (0.00/45.40/54.60) | - age, age2, sex, BMI for EMMAX-analysis - age, age2, sex, BMI, PC1, PC2, PC3, PC4 for rvtest analysis |
| **European [Finnish]** | National FINRISK 2007 Study (FINRISK 2007) | 19959603 [46] | - T2D case control study - Glucose tolerance classified according to WHO 1999 criteria - T1D and T2D (fasting plasma glucose concentration ≥7.0 mmol/l or 2-h plasma glucose concentration ≥11.1 mmol/l) cases excluded | HumanExome-12v1-1_A | >99% | - call rate ≤99% - heterozygosity >median + 3*IQR - technical duplicates with lower call rate - Non-European population outliers - sex discrepancy - contamination score >10% | ≥95% | - exclude 101 indels with different allele mapping across the two sites - exclude variants that had chromosome or position or allele mismatch - exclude duplicated variants, keeping the one with higher call rate. If the call rate is the same, arbitrarily take the one with that comes first in the file - call rate <95% - exact HWE <10^-5^ | Illumina GenCall using standard Illumina cluster files + Zcall | FG  N = 47,041 (0.00/45.12/54.88)  FI  N = 47,024 (0.00/45.12/54.88) | - age, age2, sex, BMI for EMMAX-analysis - age, age2, sex, BMI, PC1, PC2, PC3, PC4 for rvtest analysis |
| **European [Finnish]** | Finland-United States Investigation of NIDDM Genetics (FUSION) Study | 9614613 [47]; 17463248 [48] | - T2D case control study - Glucose tolerance classified according to WHO 1999 criteria - T2D (fasting plasma glucose concentration ≥7.0 mmol/l or 2-h plasma glucose concentration ≥11.1 mmol/l, by report of diabetes medication use, or based on medical record review), and known or probable T1D among their first degree relatives were excluded. | HumanExome-12v1-1_A | >99% | - call rate ≤99% - heterozygosity >median + 3*IQR - technical duplicates with lower call rate - Non-European population outliers - sex discrepancy - contamination score >10% | ≥95% | - exclude 101 indels with different allele mapping across the two sites - exclude variants that had chromosome or position or allele mismatch - exclude duplicated variants, keeping the one with higher call rate. If the call rate is the same, arbitrarily take the one with that comes first in the file - call rate <95% - exact HWE <10^-5^ | Illumina GenCall using standard Illumina cluster files + Zcall | FG  N = 56,003 (0.00/54.11/45.89)  FI  N = 55,545 (0.00/53.73/46.27) | - age, age2, sex, BMI, study origin for EMMAX-analysis - age, age2, sex, BMI, study origin, PC1, PC2, PC3, PC4 for rvtest analysis |
| **European [Finnish]** | Metabolic Syndrome in Men Study (METSIM) | 19223598 [49] | - Population-based cross-sectional study - Glucose tolerance classified according to WHO 1997 criteria - T1D and T2D (fasting plasma glucose concentration ≥7.0 mmol/l or 2-h plasma glucose concentration ≥11.1 mmol/l) cases excluded - Further excluded individuals with HbA1c ≥6.5% according to ADA 2012 criteria for T2D | HumanExome-12v1_A | >99% | - call rate ≤99% - heterozygosity >median + 3*IQR - technical duplicates with lower call rate - Non-European population outliers - sex discrepancy - contamination score >10% | ≥95% | - exclude 101 indels with different allele mapping across the two sites - exclude variants that had chromosome or position or allele mismatch - exclude duplicated variants, keeping the one with higher call rate. If the call rate is the same, arbitrarily take the one with that comes first in the file - call rate <95% - exact HWE <10^-5^ | Genotype calls generated on cluster boundaries trained on using study samples + manual review of clusterplots | FG  N = 67,415 (13.37/47.47/39.16)  FI  N = 67,414 (13.38/47.47/39.15) | - age, age2, BMI for EMMAX-analysis - age, age2, BMI, PC1, PC2, PC3, PC4 for rvtest analysis |
| **European [Danish]** | Health2006 | 23615486 [50] | - Population-based cohort - Glucose tolerance classified according to WHO 1999 criteria - T1D and T2D (fasting plasma glucose concentration ≥7.0 mmol/l) cases excluded | Illumina HumanExome-12v1 | ≥98% | - call rate <98% - heterozygosity  - sex discrepancy - discordance with previous genotypes  - Removal of population outliers | ≥98% | - exclude duplicated variants, keeping the one with higher call rate.  - call rate <95% - HWE <10^-4^ - cluster separation score 0.4 | Illumina GenCall using standard Illumina cluster files + Zcall | FG  N = 87,275 (9.43/62.52/28.06)  FI  N = 87,248 (9.43/62.50/28.07) | - age, age^2^, BMI for EMMAX-analysis - age, age^2^, BMI, PC1-10 for RareMetalWorker analysis |
| **European [Danish]** | Inter99 | 14663300 [51] | - Population-based cohort - Glucose tolerance classified according to WHO 1999 criteria - T1D and T2D (fasting plasma glucose concentration ≥7.0 mmol/l or 2-h plasma glucose concentration ≥11.1 mmol/l) cases excluded | Illumina HumanExome-12v1 | ≥98% | - call rate <98% - heterozygosity  - sex discrepancy - discordance with previous genotypes  - Removal of population outliers | ≥98% | - exclude duplicated variants, keeping the one with higher call rate.  - call rate <95% - HWE <10^-4^ - cluster separation score 0.4 | Illumina GenCall using standard Illumina cluster files + Zcall | FG  N = 97,766 (20.50/54.42/25.07)  FI  N = 96,578 (20.53/54.09/25.38) | - age, age^2^, BMI for EMMAX-analysis - age, age^2^, BMI, PC1-10 for RareMetalWorker analysis |
| **European [Danish]** | Vejle Biobank | 23160641 [52] | - Controls from T2D case-control - Glucose tolerance classified according to WHO 1999 criteria - T1D and T2D (fasting plasma glucose concentration ≥7.0 mmol/l or 2-h plasma glucose concentration ≥11.1 mmol/l) cases excluded | Illumina HumanExome-12v1 | ≥98% | - call rate <98% - heterozygosity  - sex discrepancy - discordance with previous genotypes  - Removal of population outliers | ≥98% | - exclude duplicated variants, keeping the one with higher call rate.  - call rate <95% - HWE <10^-4^ - cluster separation score 0.4 | Illumina GenCall using standard Illumina cluster files + Zcall | FG  N = 46,098 (0.00/46.88/53.12) | - age, age^2^, BMI for EMMAX-analysis - age, age^2^, BMI, PC1-10 for RareMetalWorker analysis |
| **European**  **[UK]** | Genetics of Diabetes Audit and Research Tayside (GoDARTS) | 9329309 [53] | - Population-based cohort - T2D cases, sample with fasting plasma glucose concentration ≥7.0 mmol/l and pregnant women were excluded | Illumina HumanExome-12v1_A | >99% | - call rate ≤99% - heterozygosity 4SD of mean - technical duplicates with lower call rate - Non-European population outliers  - sex discrepancy | >99% | - exclude variants that had chromosome or position or allele mismatch - exclude duplicated variants, keeping the one with higher call rate.  - call rate <98% for GenCall and <99% for zCall - exact HWE <10^-4^ - GenTrain score <0.6 and Cluster separation score <0.4 | Illumina GenCall using standard Illumina cluster files + Zcall | FG  N = 52,172 (0.00/52.63/47.37)  FI  N = 39,422 (0.00/37.25/62.75) | - age, age^2^, sex, and BMI for EMMAX-analysis - age, age^2^, sex, BMI, PC1, and PC2 for RareMetalWorker analysis |
| **European**  **[UK]** | Twins UK | 23088889 [54] | - Unrelated samples selected as controls from the Twins UK study - T1D and T2D cases and samples with recorded family history of diabetes, or if either twin was ever recorded as impaired glucose tolerant (defined as fasting plasma glucose concentration >6.1mmol/L in any reading), non-fasting were excluded. | Illumina HumanExome-12v1_A | >99% | - call rate ≤99% - heterozygosity 4SD of mean - technical duplicates with lower call rate - Non-European population outliers, or non-European reported ancestry - sex discrepancy | >99% | - exclude variants that had chromosome or position or allele mismatch - exclude duplicated variants, keeping the one with higher call rate.  - call rate <98% for GenCall and <99% for zCall - exact HWE <10^-4^ - GenTrain score <0.6 and Cluster separation score <0.4 | Illumina GenCall using standard Illumina cluster files + Zcall | FG  N = 50,643 (0.00/51.06/48.94) | - age, age^2^, sex, and BMI for EMMAX-analysis - age, age^2^, sex, BMI, PC1, and PC2 for RareMetalWorker analysis |
| **European**  **[UK]** | Oxford BioBank (OBB) | http://www.oxfordbiobank.org.uk/ | - T2D cases (on diabetic treatment or fasting glucose ≥7 mmol/l) were excluded. | Illumina HumanExome-12v1_A | >99% | - call rate ≤99% - heterozygosity 4SD of mean - technical duplicates with lower call rate - Non-European population outliers, or non-European reported ancestry - sex discrepancy | >99% | - exclude variants that had chromosome or position or allele mismatch - exclude duplicated variants, keeping the one with higher call rate.  - call rate <98% for GenCall and <99% for zCall - exact HWE <10^-4^ - GenTrain score <0.6 and Cluster separation score <0.4 | Illumina GenCall using standard Illumina cluster files + Zcall | FG  N = 86,094 (17.18/54.02/28.80)  FI  N = 83,805 (17.22/53.20/29.58) | - age, age^2^, sex, and BMI for EMMAX-analysis - age, age^2^, sex, BMI, PC1, and PC2 for RareMetalWorker analysis |
| **European [Swedish]** | Prospective Investigation of  the Vasculature in Uppsala Seniors (PIVUS) | 16141402 [55] | - Population-based cohort - T1D, T2D cases or fasting plasma glucose concentration ≥7 mmol/l, pregnant individuals, and samples with non-fasting blood excluded | Illumina HumanExome-12v1_A | >99% | - call rate ≤99% - heterozygosity 4SD of mean - technical duplicates with lower call rate - Non-European population outliers - sex discrepancy | >99% | - exclude variants that had chromosome or position or allele mismatch - exclude duplicated variants, keeping the one with higher call rate.  - call rate <98% for GenCall and <99% for zCall - exact HWE <10^-4^ - GenTrain score <0.6 and Cluster separation score <0.4 | Illumina GenCall using standard Illumina cluster files + Zcall | FG  N = 67,776 (0.00/61.67/38.33)  FI  N = 67,732 (0.00/61.68/38.32) | - age, age^2^, sex, and BMI for EMMAX-analysis - age, age^2^, sex, BMI, PC1, and PC2 for RareMetalWorker analysis |
| **European [Swedish]** | Uppsala Longitudinal Study of Adult Men (ULSAM) | 1216390 [56] | - Population-based cohort - T1D, T2D cases or fasting plasma glucose concentration ≥7 mmol/l, and samples with non-fasting blood excluded | Illumina HumanExome-12v1_A | >99% | - call rate ≤99% - heterozygosity 4SD of mean - technical duplicates with lower call rate - Non-European population outliers - sex discrepancy | >99% | - exclude variants that had chromosome or position or allele mismatch - exclude duplicated variants, keeping the one with higher call rate.  - call rate <98% for GenCall and <99% for zCall - exact HWE <10^-4^ - GenTrain score <0.6 and Cluster separation score <0.4 | Illumina GenCall using standard Illumina cluster files + Zcall | FG  N = 67,776 (0.00/61.67/38.33)  FI  N = 67,732 (0.00/61.68/38.32) | - age, age^2^, and BMI for EMMAX-analysis - age, age^2^, BMI, PC1, and PC2 for RareMetalWorker analysis |
| **European [Finnish]** | Prevalence, Prediction and Prevention of Diabetes (PPP)-Botnia study | 20454776 [57] | - Population-based cohort  - T1D, T2D cases or fasting plasma glucose concentration ≥7 mmol/l, pregnant individuals, and samples with non-fasting blood excluded | Illumina HumanExome-12v1.1 | >99% | -call rate ≤99% - heterozygosity 4SD of mean - gender discordance - GWAS discordance - genotyping platform fingerprint discordance - population outliers | >99% | - genotyping cluster checks within batches, outliers removed. - exact HWE <10^-4^ | Birdseed with cluster filter | FG  N = 67,776 (0.00/61.67/38.33)  FI  N = 67,732 (0.00/61.68/38.32) | - age, age2, and BMI for EMMAX-analysis - age, age2, sex, BMI, PC1, PC2, PC3, and PC4 for RareMetalWorker analysis |

1. Kotronen A, Yki-Jarvinen H, Mannisto S, Saarikoski L, Korpi-Hyovalti E, et al. (2010) Non-alcoholic and alcoholic fatty liver disease - two diseases of affluence associated with the metabolic syndrome and type 2 diabetes: the FIN-D2D survey. BMC Public Health 10: 237-237.
2. Tuomilehto J, Lindstrom J, Eriksson JG, Valle TT, Hamalainen H, et al. (2001) Prevention of type 2 diabetes mellitus by changes in lifestyle among subjects with impaired glucose tolerance. The New England journal of medicine 344: 1343-1350.
3. Kouki R, Schwab U, Lakka TA, Hassinen M, Savonen K, et al. (2012) Diet, fitness and metabolic syndrome--the DR's EXTRA study. Nutr Metab Cardiovasc Dis 22: 553-560.
4. Vartiainen E, Laatikainen T, Peltonen M, Juolevi A, Mannisto S, et al. (2010) Thirty-five-year trends in cardiovascular risk factors in Finland. Int J Epidemiol 39: 504-518.
5. Valle T, Tuomilehto J, Bergman RN, Ghosh S, Hauser ER, et al. (1998) Mapping genes for NIDDM. Design of the Finland-United States Investigation of NIDDM Genetics (FUSION) Study. Diabetes Care 21: 949-958.
6. Scott LJ, Mohlke KL, Bonnycastle LL, Willer CJ, Li Y, et al. (2007) A genome-wide association study of type 2 diabetes in Finns detects multiple susceptibility variants. Science 316: 1341-1345.
7. Stancakova A, Javorsky M, Kuulasmaa T, Haffner SM, Kuusisto J, et al. (2009) Changes in insulin sensitivity and insulin release in relation to glycemia and glucose tolerance in 6,414 Finnish men. Diabetes 58: 1212-1221.
8. Thuesen BH, Cerqueira C, Aadahl M, Ebstrup JF, Toft U, et al. (2013) Cohort Profile: The Health2006 cohort, Research Centre for Prevention and Health. Int J Epidemiol.
9. Jorgensen T, Borch-Johnsen K, Thomsen TF, Ibsen H, Glumer C, et al. (2003) A randomized non-pharmacological intervention study for prevention of ischaemic heart disease: baseline results Inter99. Eur J Cardiovasc Prev Rehabil 10: 377-386.
10. Albrechtsen A, Grarup N, Li Y, Sparso T, Tian G, et al. (2013) Exome sequencing-driven discovery of coding polymorphisms associated with common metabolic phenotypes. Diabetologia 56: 298-310.
11. Morris AD, Boyle DI, MacAlpine R, Emslie-Smith A, Jung RT, et al. (1997) The diabetes audit and research in Tayside Scotland (DARTS) study: electronic record linkage to create a diabetes register. DARTS/MEMO Collaboration. BMJ 315: 524-528.
12. Moayyeri A, Hammond CJ, Hart DJ, Spector TD (2013) The UK Adult Twin Registry (TwinsUK Resource). Twin Res Hum Genet 16: 144-149.
13. Lind L, Fors N, Hall J, Marttala K, Stenborg A (2005) A comparison of three different methods to evaluate endothelium-dependent vasodilation in the elderly: the Prospective Investigation of the Vasculature in Uppsala Seniors (PIVUS) study. Arterioscler Thromb Vasc Biol 25: 2368-2375.
14. Hedstrand H (1975) A study of middle-aged men with particular reference to risk factors for cardiovascular disease. Upsala journal of medical sciences Supplement 19: 1-61.
15. Isomaa B, Forsen B, Lahti K, Holmstrom N, Waden J, et al. (2010) A family history of diabetes is associated with reduced physical fitness in the Prevalence, Prediction and Prevention of Diabetes (PPP)-Botnia study. Diabetologia 53: 1709-1713.
