# Supplementary material for: Identification and Functional Characterization of G6PC2 Coding Variants Influencing Glycemic Traits Define an Effector Transcript at the G6PC2-ABCB11 Locus
Source: PLoS Genet. 2015 Jan 27;11(1):e1004876. doi: 10.1371/journal.pgen.1004876 (PMC4307976; doi:10.1371/journal.pgen.1004876)
Supplement: S2 Table — (DOCX) [file pgen.1004876.s005.docx]

|  | **Ancestry** | **European [Finnish]** | **European [Finnish]** | **European [Finnish]** | **European [Finnish]** | **European [Finnish]** | **European [Finnish]** | **European [Danish]** | **European [Danish]** | **European [Danish]** | **European**  **[UK]** | **European**  **[UK]** | **European**  **[UK]** | **European [Swedish]** | **European [Finnish]** |
| --- | --- | --- | --- | --- | --- | --- | --- | --- | --- | --- | --- | --- | --- | --- | --- |
|  | **Study** | **FIN-D2D 2007** | **The Finnish Diabetes Prevention Study (DPS)** | **The Dose Responses to Exercise Training (DR's EXTRA) Study** | **National FINRISK 2007 Study (FINRISK 2007)** | **Finland-United States Investigation of NIDDM Genetics (FUSION) Study** | **Metabolic Syndrome in Men  (METSIM)** | **Health2006** | **Inter99** | **Vejle Biobank** | **Genetics of Diabetes Audit and Research Tayside (GoDARTS)** | **Twins UK** | **Oxford BioBank (OBB)** | **Pivus and Ulsam** | **Prevalence, Prediction and Prevention of Diabetes (PPP)-Botnia study** |
| **Single variant analysis (relateds)** | # available | 2132 | 328 | 622 | 549 | 1414 | 6599 | 3329 | 5431 | 435 | 802 | 701 | 4513 | 1859 | 4533 |
|  | Mean fasting glucose (SD), mmol/l | F: 5.79 (0.45) | F: 5.86 (0.59) | F: 5.45 (0.47) | F: 5.58 (0.30) | F: 5.22 (0.46) | F: 0 (0) | F: 5.31 (0.52) | F: 5.28 (0.47) | F: 5.07 (0.32) | F: 4.81 (0.45) | F: 4.71 (0.59) | F: 5.11 (0.41) | F: 4.96 (0.56) | F: 5.24 (0.54) |
|  |  | M: 6.04 (0.43) | M: 5.93 (0.54) | M: 5.68 (0.48) | M: 5.71 (0.27) | M: 5.37 (0.49) | M: 5.72 (0.48) | M: 5.57 (0.51) | M: 5.58 (0.48) | M: 5.19 (0.3) | M: 4.98 (0.48) | M: 4.88 (0.64) | M: 5.41 (0.45) | M: 5.26 (0.58) | M: 5.29 (0.56) |
|  | # Females (%) | 1154 (54.13) | 224 (68.29) | 448 (72.03) | 328 (59.74) | 653 (46.18) | 0 (0.0) | 1437 (43.17) | 2560 (47.14) | 147 (33.79) | 369 (46.01) | 582 (83.02) | 2474 (54.82) | 444 (23.88) | 2448 (0.54) |
|  | Mean age (SD),  Years | F: 58.76 (8.23) | F: 55.06 (7.19) | F: 66.29 (5.22) | F: 63.97 (7.53) | F: 62.65 (7.70) | F: 0 (0) | F: 47.93 (12.3) | F: 45.42 (7.92) | F: 55.13 (12.52) | F: 57.07 (11.09) | F: 44.13 (12.98) | F: 41.57 (5.98) | F: 70.26 (0.15) | F: 48.73 (15.67) |
|  |  | M: 59.49 (8.44) | M: 56.10 (7.16) | M: 67.90 (6.38) | M: 65.64 (6.49) | M: 60.40 (8.11) | M: 57.40 (7.24) | M: 48.82 (12.12) | M: 45.8 (7.82) | M: 62.36 (10.21) | M: 58.95 (10.80) | M: 45.18 (12.06) | M: 42.04 (5.62) | M: 70.72 (0.67) | M: 48.43 (15.56) |
|  | Mean BMI (SD), kg/m^2^ | F: 27.00 (4.96) | F: 31.78 (4.88) | F: 27.23 (4.66) | F: 30.08 (4.91) | F: 26.52 (4.24) | F: 0 (0) | F: 25.12 (4.69) | F: 25.47 (4.78) | F: 21.75 (1.93) | F: 26.61 (4.78) | F: 23.75 (4.01) | F: 25.44 (4.80) | F: 26.92 (4.70) | F: 25.85 (4.656) |
|  |  | M: 26.81 (3.74) | M: 29.54 (3.46) | M: 27.25 (3.59) | M: 28.59 (3.85) | M: 26.56 (3.49) | M: 26.89 (3.83) | M: 26.37 (3.81) | M: 26.58 (3.84) | M: 23.05 (1.47) | M: 27.59 (4.05) | M: 23.81 (3.85) | M: 26.61 (4.00) | M: 26.17 (3.37) | M: 26.66 (3.75) |
|  | # available (with BMI) | 2128 | 328 | 622 | 549 | 1414 | 6597 | 3327 | 5427 | 435 | 801 | 701 | 4513 | 1857 | 4532 |
|  | Mean fasting glucose (SD), mmol/l | F: 5.79 (0.45) | F: 5.86 (0.59) | F: 5.45 (0.47) | F: 5.58 (0.30) | F: 5.22 (0.46) | F: 0 (0) | F: 5.31 (0.52) | F: 5.28 (0.47) | F: 5.07 (0.32) | F: 4.81 (0.45) | F: 4.71 (0.59) | F: 5.11 (0.41) | F: 4.96 (0.56) | F: 5.24 (0.54) |
|  |  | M: 6.04 (0.42) | M: 5.93 (0.54) | M: 5.68 (0.48) | M: 5.71 (0.27) | M: 5.37 (0.49) | M: 5.72 (0.48) | M: 5.57 (0.51) | M: 5.58 (0.48) | M: 5.19 (0.3) | M: 4.98 (0.48) | M: 4.88 (0.64) | M: 5.41 (0.45) | M: 5.26 (0.58) | M: 5.29 (0.56) |
|  | # Females (%) | 1152 (54.14) | 224 (68.29) | 448 (72.03) | 328 (59.74) | 653 (46.18) | 0 (0.0) | 1437 (43.19) | 2559 (47.15) | 147 (33.79) | 368 (45.94) | 582 (83.02) | 2474 (54.82) | 444 (23.91) | 2447 (0.54) |
|  | Mean age (SD),  Years | F: 58.77 (8.23) | F: 55.06 (7.19) | F: 66.29 (5.22) | F: 63.97 (7.53) | F: 62.65 (7.70) | F: 0 (0) | F: 47.94 (12.3) | F: 45.43 (7.92) | F: 55.13 (12.52) | F: 57.04 (11.09) | F: 44.13 (12.98) | F: 41.57 (5.98) | F: 70.26 (0.15) | F: 48.73 (15.67) |
|  |  | M: 59.47 (8.43) | M: 56.10 (7.16) | M: 67.90 (6.38) | M: 65.64 (6.49) | M: 60.40 (8.11) | M: 57.40 (7.24) | M: 48.82 (12.12) | M: 45.81 (7.82) | M: 62.36 (10.21) | M: 58.95 (10.80) | M: 45.18 (12.06) | M: 42.04 (5.62) | M: 70.72 (0.67) | M: 48.43 (15.56) |
|  | Mean BMI (SD), kg/m^2^ | F: 27.00 (4.96) | F: 31.78 (4.88) | F: 27.23 (4.66) | F: 30.08 (4.91) | F: 26.52 (4.24) | F: 0 (0) | F: 25.12 (4.69) | F: 25.47 (4.78) | F: 21.75 (1.93) | F: 26.68 (4.78) | F: 23.75 (4.01) | F: 25.44 (4.80) | F: 26.92 (4.70) | F: 25.85 (4.656) |
|  |  | M: 26.81 (3.74) | M: 29.54 (3.46) | M: 27.25 (3.59) | M: 28.59 (3.85) | M: 26.56 (3.49) | M: 26.89 (3.83) | M: 26.37 (3.81) | M: 26.58 (3.84) | M: 23.05 (1.47) | M: 27.59 (4.05) | M: 23.81 (3.85) | M: 26.61 (4.00) | M: 26.21 (3.37) | M: 26.66 (3.75) |
|  | # available | 2111 | 306 | 657 | 548 | 1342 | 6596 | 3328 | 5227 | 0 | 244 | 0 | 4136 | 1853 | 4492 |
|  | Mean fasting insulin (SD), pmol/l | F: 40.90 (20.63) | F: 82.99 (41.26) | F: 41.66 (25.96) | F: 40.74 (21.64) | F: 52.18 (33.05) | F: 0 (0) | F: 38.02 (24.97) | F: 38.65 (25.03) | NA | F: 64.15 (31.44) | NA | F: 94.71 (39.61) | F: 58.00 (35.62) | F: 8.46 (6.6) |
|  |  | M: 43.84 (26.76) | M: 84.39 (42.06) | M: 44.82 (32.97) | M: 37.02 (19.73) | M: 49.27 (30.38) | M: 50.70 (36.47) | M: 40.61 (27.58) | M: 42.53 (27.71) | NA | M: 80.97 (55.91) | NA | M: 105.54 (51.97) | M: 77.45 (45.29) | M: 9.16 (7.5) |
|  | # Females (%) | 1141 (54.05) | 214 (69.93) | 472 (71.84) | 327 (59.67) | 632 (47.09) | 0 (0.0) | 1436 (43.15) | 2474 (47.33) | NA | 127 (52.05) | NA | 2240 (54.16) | 445 (24.02) | 2426 (0.54) |
|  | Mean age (SD),  Years | F: 58.75 (8.23) | F: 54.98 (7.09) | F: 66.33 (5.21) | F: 64.02 (7.48) | F: 62.71 (7.80) | F: 0 (0) | F: 47.93 (12.3) | F: 45.37 (7.93) | NA | F: 56.40 (10.82) | NA | F: 41.65 (5.99) | F: 70.26 (0.15) | F: 48.69 (15.68) |
|  |  | M: 59.49 (8.46) | M: 55.77 (7.27) | M: 67.89 (6.34) | M: 65.64 (6.49) | M: 60.70 (8.11) | M: 57.40 (7.24) | M: 48.82 (12.12) | M: 45.78 (7.8) | NA | M: 60.04 (9.25) | NA | M: 42.11 (5.63) | M: 70.72 (0.67) | M: 48.39 (15.53) |
|  | Mean BMI (SD), kg/m^2^ | F: 27.01 (4.97) | F: 31.92 (4.89) | F: 27.19 (4.66) | F: 30.05 (4.89) | F: 26.52 (4.23) | F: 0 (0) | F: 25.12 (4.69) | F: 25.51 (4.8) | NA | F: 25.66 (4.29) | NA | F: 25.46 (4.75) | F: 26.92 (4.69) | F: 25.85 (4.663) |
|  |  | M: 26.79 (3.73) | M: 29.64 (3.47) | M: 27.10 (3.58) | M: 28.59 (3.85) | M: 26.51 (3.42) | M: 26.89 (3.83) | M: 26.37 (3.81) | M: 26.58 (3.85) | NA | M: 27.36 (3.02) | NA | M: 26.58 (3.97) | M: 26.18 (3.38) | M: 26.67 (3.759) |
|  | # available (with BMI) | 2107 | 306 | 657 | 548 | 1342 | 6594 | 3326 | 5223 | 0 | 244 | NA | 4136 | 1851 | 4491 |
|  | Mean fasting insulin (SD), pmol/l | F: 40.91 (20.65) | F: 82.99 (41.26) | F: 41.66 (25.96) | F: 40.74 (21.64) | F: 52.18 (33.05) | F: 0 (0) | F: 38.01 (24.97) | F: 38.62 (25.01) | NA | F: 64.15 (31.44) | NA | F: 94.71 (39.61) | F: 58.00 (35.62) | F: 8.46 (6.6) |
|  |  | M: 43.79 (26.74) | M: 84.39 (42.06) | M: 44.82 (32.97) | M: 37.02 (19.73) | M: 49.27 (30.38) | M: 50.70 (36.47) | M: 40.61 (27.58) | M: 42.54 (27.72) | NA | M: 80.97 (55.91) | NA | M: 105.54 (51.97) | M: 77.40 (45.19) | M: 9.16 (7.5) |
|  | # Females (%) | 1139 (54.06) | 214 (69.93) | 472 (71.84) | 327 (59.67) | 632 (47.09) | 0 (0.0) | 1436 (43.17) | 2473 (47.35) | NA | 127 (52.05) | NA | 2240 (54.16) | 445 (24.04) | 2425 (0.54) |
|  | Mean age (SD),  years | F: 58.77 (8.23) | F: 54.98 (7.09) | F: 66.33 (5.21) | F: 64.02 (7.48) | F: 62.71 (7.80) | F: 0 (0) | F: 47.94 (12.3) | F: 45.38 (7.93) | NA | F: 56.40 (10.82) | NA | F: 41.65 (5.99) | F: 70.26 (0.15) | F: 48.69 (15.69) |
|  |  | M: 59.47 (8.45) | M: 55.77 (7.27) | M: 67.89 (6.34) | M: 65.64 (6.49) | M: 60.70 (8.11) | M: 57.40 (7.24) | M: 48.82 (12.12) | M: 45.79 (7.79) | NA | M: 60.04 (9.25) | NA | M: 42.11 (5.63) | M: 70.72 (0.67) | M: 48.39 (15.53) |
|  | Mean BMI (SD), kg/m^2^ | F: 27.01 (4.97) | F: 31.92 (4.89) | F: 27.19 (4.66) | F: 30.05 (4.89) | F: 26.52 (4.23) | F: 0 (0) | F: 25.12 (4.69) | F: 25.51 (4.8) | NA | F: 25.66 (4.29) | NA | F: 25.46 (4.75) | F: 26.92 (4.69) | F: 25.85 (4.663) |
|  |  | M: 26.79 (3.73) | M: 29.64 (3.47) | M: 27.10 (3.58) | M: 28.59 (3.85) | M: 26.51 (3.42) | M: 26.89 (3.83) | M: 26.37 (3.81) | M: 26.58 (3.85) | NA | M: 27.36 (3.02) | NA | M: 26.58 (3.97) | M: 26.22 (3.38) | M: 26.67 (3.759) |
| **Gene-level analysis (unrelateds)** | # available | 2132 | 328 | 622 | 549 | 1414 | 6599 | 3214 | 5279 | 430 | 801 | 697 | 4442 | 1804 | 3932 |
|  | Mean fasting glucose (SD), mmol/l | F: 5.79 (0.45) | F: 5.86 (0.59) | F: 5.45 (0.47) | F: 5.58 (0.30) | F: 5.22 (0.46) | F: 0 (0) | F: 5.31 (0.52) | F: 5.28 (0.47) | F: 5.08 (0.32) | F: 4.81 (0.45) | F: 4.71 (0.59) | F: 5.11 (0.41) | F: 4.96 (0.56) | F: 5.24 (0.55) |
|  |  | M: 6.04 (0.43) | M: 5.93 (0.54) | M: 5.68 (0.48) | M: 5.71 (0.27) | M: 5.37 (0.49) | M: 5.72 (0.48) | M: 5.58 (0.51) | M: 5.58 (0.48) | M: 5.19 (0.3) | M: 4.98 (0.48) | M: 4.87 (0.64) | M: 5.41 (0.45) | M: 5.26 (0.58) | M: 5.29 (0.56) |
|  | # Females (%) | 1154 (54.13) | 224 (68.29) | 448 (72.03) | 328 (59.74) | 653 (46.18) | 0 (0.0) | 1393 (43.34) | 2486 (47.09) | 146 (33.95) | 369 (46.07) | 580 (83.21) | 2430 (54.71) | 437 (24.22) | 2161 (0.55) |
|  | Mean age (SD),  years | F: 58.76 (8.23) | F: 55.06 (7.19) | F: 66.29 (5.22) | F: 63.97 (7.53) | F: 62.65 (7.70) | F: 0 (0) | F: 47.92 (12.24) | F: 45.43 (7.92) | F: 55.19 (12.6) | F: 57.07 (11.09) | F: 44.19 (12.95) | F: 41.57 (5.99) | F: 70.26 (0.15) | F: 48.66 (15.57) |
|  |  | M: 59.49 (8.44) | M: 56.10 (7.16) | M: 67.90 (6.38) | M: 65.64 (6.49) | M: 60.40 (8.11) | M: 57.40 (7.24) | M: 48.95 (12.07) | M: 45.78 (7.79) | M: 62.3 (10.22) | M: 58.92 (10.80) | M: 45.08 (12.12) | M: 42.09 (5.61) | M: 70.72 (0.67) | M: 48.28 (15.64) |
|  | Mean BMI (SD), kg/m^2^ | F: 27.00 (4.96) | F: 31.78 (4.88) | F: 27.23 (4.66) | F: 30.08 (4.91) | F: 26.52 (4.24) | F: 0 (0) | F: 25.13 (4.73) | F: 25.46 (4.78) | F: 21.74 (1.93) | F: 26.61 (4.78) | F: 23.76 (4.01) | F: 25.43 (4.81) | F: 26.93 (4.71) | F: 25.85 (4.636) |
|  |  | M: 26.81 (3.74) | M: 29.54 (3.46) | M: 27.25 (3.59) | M: 28.59 (3.85) | M: 26.56 (3.49) | M: 26.89 (3.83) | M: 26.4 (3.84) | M: 26.56 (3.81) | M: 23.04 (1.47) | M: 27.60 (4.05) | M: 23.79 (3.82) | M: 26.60 (4.00) | M: 26.18 (3.40) | M: 26.59 (3.73) |
|  | # available (with BMI) | 2128 | 328 | 622 | 549 | 1414 | 6597 | 3212 | 5275 | 430 | 800 | 697 | 4442 | 1802 | 3931 |
|  | Mean fasting glucose (SD), mmol/l | F: 5.79 (0.45) | F: 5.86 (0.59) | F: 5.45 (0.47) | F: 5.58 (0.30) | F: 5.22 (0.46) | F: 0 (0) | F: 5.31 (0.52) | F: 5.28 (0.47) | F: 5.08 (0.32) | F: 4.81 (0.45) | F: 4.71 (0.59) | F: 5.11 (0.41) | F: 4.96 (0.56) | F: 5.24 (0.55) |
|  |  | M: 6.04 (0.42) | M: 5.93 (0.54) | M: 5.68 (0.48) | M: 5.71 (0.27) | M: 5.37 (0.49) | M: 5.72 (0.48) | M: 5.58 (0.51) | M: 5.58 (0.48) | M: 5.19 (0.3) | M: 4.98 (0.48) | M: 4.87 (0.64) | M: 5.41 (0.45) | M: 5.26 (0.58) | M: 5.29 (0.56) |
|  | # Females (%) | 1152 (54.14) | 224 (68.29) | 448 (72.03) | 328 (59.74) | 653 (46.18) | 0 (0.0) | 1393 (43.37) | 2485 (47.11) | 146 (33.95) | 368 (46.00) | 580 (83.21) | 2430 (54.71) | 437 (24.25) | 2160 (0.55) |
|  | Mean age (SD),  years | F: 58.77 (8.23) | F: 55.06 (7.19) | F: 66.29 (5.22) | F: 63.97 (7.53) | F: 62.65 (7.70) | F: 0 (0) | F: 47.94 (12.23) | F: 45.44 (7.91) | F: 55.19 (12.6) | F: 57.04 (11.09) | F: 44.19 (12.95) | F: 41.57 (5.99) | F: 70.26 (0.15) | F: 48.66 (15.57) |
|  |  | M: 59.47 (8.43) | M: 56.10 (7.16) | M: 67.90 (6.38) | M: 65.64 (6.49) | M: 60.40 (8.11) | M: 57.40 (7.24) | M: 48.95 (12.07) | M: 45.79 (7.79) | M: 62.3 (10.22) | M: 58.92 (10.80) | M: 45.08 (12.12) | M: 42.09 (5.61) | M: 70.72 (0.67) | M: 48.28 (15.64) |
|  | Mean BMI (SD), kg/m^2^ | F: 27.00 (4.96) | F: 31.78 (4.88) | F: 27.23 (4.66) | F: 30.08 (4.91) | F: 26.52 (4.24) | F: 0 (0) | F: 25.13 (4.73) | F: 25.46 (4.78) | F: 21.74 (1.93) | F: 26.68 (4.78) | F: 23.76 (4.01) | F: 25.43 (4.81) | F: 26.93 (4.71) | F: 25.85 (4.66) |
|  |  | M: 26.81 (3.74) | M: 29.54 (3.46) | M: 27.25 (3.59) | M: 28.59 (3.85) | M: 26.56 (3.49) | M: 26.89 (3.83) | M: 26.4 (3.84) | M: 26.56 (3.81) | M: 23.04 (1.47) | M: 27.60 (4.05) | M: 23.79 (3.82) | M: 26.60 (4.00) | M: 26.22 (3.40) | M: 26.59 (3.73) |
|  | # available | 2111 | 306 | 657 | 548 | 1342 | 6596 | 3213 | 5076 | 0 | 244 | 0 | 4075 | 1799 | 3898 |
|  | Mean fasting insulin (SD), pmol/l | F: 40.90 (20.63) | F: 82.99 (41.26) | F: 41.66 (25.96) | F: 40.74 (21.64) | F: 52.18 (33.05) | F: 0 (0) | F: 38.02 (25.02) | F: 38.57 (24.84) | NA | F: 64.15 (31.44) | NA | F: 94.69 (39.65) | F: 58.06 (35.82) | F: 8.41 (6.1) |
|  |  | M: 43.84 (26.76) | M: 84.39 (42.06) | M: 44.82 (32.97) | M: 37.02 (19.73) | M: 49.27 (30.38) | M: 50.70 (36.47) | M: 40.87 (27.84) | M: 42.33 (27.51) | NA | M: 80.97 (55.91) | NA | M: 105.55 (51.87) | M: 77.50 (45.65) | M: 9.04 (7.1) |
|  | # Females (%) | 1141 (54.05) | 214 (69.93) | 472 (71.84) | 327 (59.67) | 632 (47.09) | 0 (0.0) | 1392 (43.32) | 2401 (47.3) | NA | 127 (52.05) | NA | 2204 (54.09) | 438 (24.35) | 2142 (0.55) |
|  | Mean age (SD),  years | F: 58.75 (8.23) | F: 54.98 (7.09) | F: 66.33 (5.21) | F: 64.02 (7.48) | F: 62.71 (7.80) | F: 0 (0) | F: 47.92 (12.24) | F: 45.38 (7.93) | NA | F: 56.40 (10.82) | NA | F: 41.64 (6.00) | F: 70.26 (0.15) | F: 48.62 (15.59) |
|  |  | M: 59.49 (8.46) | M: 55.77 (7.27) | M: 67.89 (6.34) | M: 65.64 (6.49) | M: 60.70 (8.11) | M: 57.40 (7.24) | M: 48.95 (12.08) | M: 45.76 (7.77) | NA | M: 60.04 (9.25) | NA | M: 42.15 (5.63) | M: 70.72 (0.67) | M: 48.25 (15.61) |
|  | Mean BMI (SD), kg/m^2^ | F: 27.01 (4.97) | F: 31.92 (4.89) | F: 27.19 (4.66) | F: 30.05 (4.89) | F: 26.52 (4.23) | F: 0 (0) | F: 25.13 (4.73) | F: 25.5 (4.8) | NA | F: 25.66 (4.29) | NA | F: 25.45 (4.77) | F: 26.94 (4.71) | F: 25.86 (4.67) |
|  |  | M: 26.79 (3.73) | M: 29.64 (3.47) | M: 27.10 (3.58) | M: 28.59 (3.85) | M: 26.51 (3.42) | M: 26.89 (3.83) | M: 26.4 (3.84) | M: 26.56 (3.83) | NA | M: 27.36 (3.02) | NA | M: 26.58 (3.97) | M: 26.19 (3.41) | M: 26.59 (3.74) |
|  | # available (with BMI) | 2107 | 306 | 657 | 548 | 1342 | 6594 | 3211 | 5072 | 0 | 244 | NA | 4075 | 1797 | 3897 |
|  | Mean fasting insulin (SD), pmol/l | F: 40.91 (20.65) | F: 82.99 (41.26) | F: 41.66 (25.96) | F: 40.74 (21.64) | F: 52.18 (33.05) | F: 0 (0) | F: 38.01 (25.03) | F: 38.54 (24.82) | NA | F: 64.15 (31.44) | NA | F: 94.69 (39.65) | F: 58.06 (35.82) | F: 8.42 (6.1) |
|  |  | M: 43.79 (26.74) | M: 84.39 (42.06) | M: 44.82 (32.97) | M: 37.02 (19.73) | M: 49.27 (30.38) | M: 50.70 (36.47) | M: 40.87 (27.84) | M: 42.33 (27.51) | NA | M: 80.97 (55.91) | NA | M: 105.55 (51.87) | M: 77.45 (45.55) | M: 9.02 (7.1) |
|  | # Females (%) | 1139 (54.06) | 214 (69.93) | 472 (71.84) | 327 (59.67) | 632 (47.09) | 0 (0.0) | 1392 (43.35) | 2400 (47.32) | NA | 127 (52.05) | NA | 2204 (54.09) | 438 (24.37) | 2141 (0.55) |
|  | Mean age (SD),  years | F: 58.77 (8.23) | F: 54.98 (7.09) | F: 66.33 (5.21) | F: 64.02 (7.48) | F: 62.71 (7.80) | F: 0 (0) | F: 47.94 (12.23) | F: 45.39 (7.93) | NA | F: 56.40 (10.82) | NA | F: 41.64 (6.00) | F: 70.26 (0.15) | F: 48.61 (15.59) |
|  |  | M: 59.47 (8.45) | M: 55.77 (7.27) | M: 67.89 (6.34) | M: 65.64 (6.49) | M: 60.70 (8.11) | M: 57.40 (7.24) | M: 48.95 (12.08) | M: 45.77 (7.76) | NA | M: 60.04 (9.25) | NA | M: 42.15 (5.63) | M: 70.72 (0.67) | M: 48.25 (15.61) |
|  | Mean BMI (SD), kg/m^2^ | F: 27.01 (4.97) | F: 31.92 (4.89) | F: 27.19 (4.66) | F: 30.05 (4.89) | F: 26.52 (4.23) | F: 0 (0) | F: 25.13 (4.73) | F: 25.5 (4.8) | NA | F: 25.66 (4.29) | NA | F: 25.45 (4.77) | F: 26.94 (4.71) | F: 25.86 (4.67) |
|  |  | M: 26.79 (3.73) | M: 29.64 (3.47) | M: 27.10 (3.58) | M: 28.59 (3.85) | M: 26.51 (3.42) | M: 26.89 (3.83) | M: 26.4 (3.84) | M: 26.56 (3.83) | NA | M: 27.36 (3.02) | NA | M: 26.58 (3.97) | M: 26.23 (3.41) | M: 26.59 (3.74) |
