# Supplementary material for: Identification and Functional Characterization of G6PC2 Coding Variants Influencing Glycemic Traits Define an Effector Transcript at the G6PC2-ABCB11 Locus
Source: PLoS Genet. 2015 Jan 27;11(1):e1004876. doi: 10.1371/journal.pgen.1004876 (PMC4307976; doi:10.1371/journal.pgen.1004876)
Supplement: S3 Table — MAF: minor allele frequency. β^: regression coefficient estimates. SE: standard error. N: number of samples analyzed. I2: heterogeneity measure in %. P_het: P-value for Cochran’s Q statistic. aSample-size weighted average minor allele frequency percentage across all studies. (DOCX) [file pgen.1004876.s006.docx]

| **Locus** | **SNP** | **Minor/Major Allele** | **MAF^a^ (%)** | **Variant** | **Gene** | **Trait** | ***P*** | $\hat{\boldsymbol{\beta}}$  **(SE)** | **N** | **I^2^** | **Cochran’s Q** | ***P*_het** | **Conditioning**  **variant** | ***P* conditional** |
| --- | --- | --- | --- | --- | --- | --- | --- | --- | --- | --- | --- | --- | --- | --- |
| **Coding variants within known GWAS regions** | | | | | | | | | | | | | | |
| *GCKR* | rs1260326 | T/C | 36.9 | p.Pro446Leu | *GCKR* | FG | 5.3x10^-18^ | -0.031 (0.004) | 33,230 | 31.7 | 19.04 | 0.12 | - | - |
|  |  |  |  |  |  | FI | 8.1x10^-11^ | -0.023 (0.004) | 30,824 | 0 | 8.69 | 0.65 | - | - |
|  | rs1919128 | G/A | 26.2 | p.Ile774Val | *C2orf16* | FG | 3.5x10^-9^ | -0.023 (0.004) | 33,231 | 0 | 10.61 | 0.64 | rs1260326 | 0.94 |
|  |  |  |  |  |  | FI | 1.5x10^-8^ | -0.022 (0.004) | 30,825 | 43.2 | 19.38 | 0.055 | rs1260326 | 0.16 |
|  | rs3749147 | A/G | 24.4 | p.Arg12Lys | *GPN1* | FG | 6.3x10^-8^ | -0.022 (0.004) | 28,655 | 0 | 9.076 | 0.69 | rs1260326 | 0.39 |
|  |  |  |  |  |  | FI | 1.2x10^-7^ | -0.022 (0.005) | 26,290 | 32.6 | 14.85 | 0.14 | rs1260326 | 0.22 |
|  | rs1395 | G/A | 29.6 | p.Ser481Phe | *SLC5A6* | FG | 1.2x10^-7^ | 0.021 (0.004) | 33,230 | 25.7 | 17.51 | 0.18 | rs1260326 | 1.00 |
| *SLC30A8* | rs13266634 | T/C | 35.7 | p.Arg325Trp | *SLC30A8* | FG | 2.5x10^-10^ | -0.023 (0.004) | 33,230 | 0 | 12.71 | 0.47 | - | - |
| *G6PC2*/*ABCB11* | rs492594 | C/G | 48.1 | p.Val219Leu | *G6PC2* | FG | 6.0x10^-9^ | 0.020 (0.004) | 33,231 | 0 | 6.68 | 0.92 | rs560887 | 7.1x10^-10^ |
|  | rs138726309 | T/C | 0.8 | p.His177Tyr | *G6PC2* | FG | 3.1x10^-8^ | -0.102 (0.020) | 32,430 | 27.2 | 16.49 | 0.17 | rs560887 | 1.3x10^-11^ |
| *RREB1* | rs35742417 | A/C | 21.1 | p.Ser1554Tyr | *RREB1* | FG | 8.4x10^-9^ | -0.024 (0.004) | 33,230 | 0 | 12.28 | 0.51 | - | - |
| *PCSK1* | rs6235 | G/C | 27.9 | p.Ser690Thr | *PCSK1* | FG | 4.1x10^-8^ | -0.022 (0.004) | 33,231 | 0 | 8.65 | 0.80 | rs4869272 | 0.01 |
|  | rs6234 | C/G | 27.9 | p.Gln665Glu | *PCSK1* | FG | 3.0x10^-8^ | -0.022 (0.004) | 33,231 | 0 | 8.58 | 0.80 | rs4869272 | 0.0081 |
| *COBLL1* | rs7607980 | C/T | 11.3 | p.Asn939Asp | *COBLL1* | FI | 6.7x10^-8^ | -0.030 (0.006) | 30,825 | 0 | 10.90 | 0.45 | - | - |
| *TOP1* | rs17265513 | C/T | 23.8 | p.Asn310Ser | *ZHX3* | FG | 3.9x10^-7^ | 0.022 (0.004) | 33,229 | 0 | 12.01 | 0.53 | rs6072275 | 0.052 |
| *PPARG* | rs1801282 | G/C | 14.7 | p.Pro12Ala | *PPARG* | FI | 1.3x10^-7^ | -0.025 (0.004) | 30,824 | 33.6 | 16.58 | 0.12 | - | - |
| **Coding variants outside known GWAS regions** | | | | | | | | | | | | | | |
| *GLP1R* | rs10305492 | A/G | 1.5 | p.Ala316Thr | *GLP1R* | FG | 4.6x10^-7^ | -0.073 (0.015) | 33,230 | 0 | 6.32 | 0.85 | - | - |
| *URB2* | rs141203811 | T/A | 0.1 | p.Glu594Val | *URB2* | FI | 3.1x10^-7^ | 0.282 (0.066) | 21,130 | 59.9 | 9.98 | 0.04 | - | - |
