# Supplementary material for: Identification and Functional Characterization of G6PC2 Coding Variants Influencing Glycemic Traits Define an Effector Transcript at the G6PC2-ABCB11 Locus
Source: PLoS Genet. 2015 Jan 27;11(1):e1004876. doi: 10.1371/journal.pgen.1004876 (PMC4307976; doi:10.1371/journal.pgen.1004876)
Supplement: S4 Table — GERP: Genomic Evolutionary Rate Profiling (DOCX) [file pgen.1004876.s007.docx]

| **SNP** | **Gene** | **Protein change** | **SIFT** | **PolyPhen-2** | **Condel** | **GERP** |
| --- | --- | --- | --- | --- | --- | --- |
| rs10305492 | *GLP1R* | p.Ala316Thr | Tolerated | Possibly damaging | Neutral | 4.80 |
| rs138726309 | *G6PC2* | p.His177Tyr | Deleterious | Possibly damaging | Deleterious | 5.90 |
| rs492594 | *G6PC2* | p.Val219Leu | Tolerated | Benign | Neutral | 3.06 |
| rs2232323 | *G6PC2* | p.Tyr207Ser | Deleterious | Probably damaging | Deleterious | 4.73 |
| rs145050507 | *G6PC2* | p.Ile171Tyr | Deleterious | Benign | Neutral | 4.73 |
| rs2232326 | *G6PC2* | p.Ser324Pro | Deleterious | Possibly damaging | Deleterious | 5.76 |
| rs6234 | *PCSK1* | p.Gln665Glu | Tolerated | Benign | Neutral | -0.27 |
| rs6235 | *PCSK1* | p.Ser690Thr | Tolerated | Benign | Neutral | 3.95 |
| rs17265513 | *ZHX3* | p.Asn310Ser | Deleterious | Possibly damaging | Deleterious | 4.79 |
| rs35742417 | *RREB1* | p.Ser1554Tyr | Tolerated | Benign | Neutral | 3.42 |
| rs141203811 | *URB2* | p.Glu594Val | Tolerated | Benign | Neutral | -3.73 |
