# Supplementary material for: Identification and Functional Characterization of G6PC2 Coding Variants Influencing Glycemic Traits Define an Effector Transcript at the G6PC2-ABCB11 Locus
Source: PLoS Genet. 2015 Jan 27;11(1):e1004876. doi: 10.1371/journal.pgen.1004876 (PMC4307976; doi:10.1371/journal.pgen.1004876)
Supplement: S5 Table — * Although AKT2 reached exome-wide significance, the signal was driven by a single variant which showed only suggestive significance in single-variant analysis (P=9.3×10^−7^). As described in the methods, we required the single-variant test to be exome-wide significant (P<5×10^−7^) in such a scenario (DOCX) [file pgen.1004876.s008.docx]

| **Gene** |  | ***P*_SKAT_** | ***P*_BURDEN_** |
| --- | --- | --- | --- |
| ***FG*** | | | |
| ***G6PC2*** | **Unconditional for all 15 variants in PTV + missense** | 3.6x10^-12^ | 5.1x10^-13^ |
|  | - **Conditioning on** p.His177Tyr (rs138726309) - **Conditioning on** p.Tyr207Ser (rs2232323) - **Conditioning on** p.His177Tyr, and p.Tyr207Ser - **Conditioning on** p.His177Tyr, p.Tyr207Ser, and p.Ile171Thr (rs145050507) - **Conditioning on** p.His177Tyr, p.Tyr207Ser, and p.Ser324Pro (rs2232326) - **Conditioning on** p.His177Tyr, p.Tyr207Ser, p.Ile171Thr, and p.Ser324Pro | 1.6x10^-8^  2.2x10^-9^  4.8x10^-5^  0.0024  0.0034  0.35 | 1.0x10^-9^  5.1x10^-11^  0.00019  0.012  0.015  0.39 |
|  | **Unconditional for all 4 variants PTV + NS_strict_** | 3.6x10^-12^ | 5.1x10^-13^ |
|  | - **Conditioning on** p.His177Tyr (rs138726309) - **Conditioning on** p.Tyr207Ser (rs2232323) - **Conditioning on** p.His177Tyr, and p.Tyr207Ser | 7.0x10^-7^  3.9x10^-8^  0.58 | 1.6x10^-6^  7.3x10^-8^  0.44 |
|  | **Unconditional for all 12 variants PTV + NS_broad_** | 2.0x10^-13^ | 1.2x10^-17^ |
|  | - **Conditioning on** p.His177Tyr (rs138726309) - **Conditioning on** p.Tyr207Ser (rs2232323) - **Conditioning on** p.His177Tyr, and p.Tyr207Ser - **Conditioning on** p.His177Tyr, p.Tyr207Ser, and p.Ile171Thr (rs145050507) - **Conditioning on** p.His177Tyr, p.Tyr207Ser, and p.Ser324Pro (rs2232326) - **Conditioning on** p.His177Tyr, p.Tyr207Ser, p.Ile171Thr, and p.Ser324Pro | 1.9x10^-8^  2.5x10^-9^  4.5x10^-5^  0.0017  0.0026  0.27 | 2.9x10^-11^  1.8x10^-12^  7.4x10^-6^  0.00089  0.0015  0.10 |
| **FI** | | | |
| ***AKT2**** | **Unconditional for all 3 variants PTV + NS_strict_** | 9.2x10^-7^ | 2.3x10^-6^ |
|  | - **Conditioning on** p.Pro50Thr (rs184042322) | 0.47 | 0.43 |
