# Supplementary material for: Identification and Functional Characterization of G6PC2 Coding Variants Influencing Glycemic Traits Define an Effector Transcript at the G6PC2-ABCB11 Locus
Source: PLoS Genet. 2015 Jan 27;11(1):e1004876. doi: 10.1371/journal.pgen.1004876 (PMC4307976; doi:10.1371/journal.pgen.1004876)
Supplement: S6 Table — MAF: minor allele frequency; Allele counts: Minor allele counts. aAlleles are aligned to the forward strand of NCBI Build 37. bSample-size weighted average minor allele frequency percentage across all studies. P values are obtained with derived inverse normalized residuals of mmol/L of fasting glucose after adjustment for age, sex, and BMI. Direction of effect is aligned to the minor allele. (DOCX) [file pgen.1004876.s009.docx]

|  |  |  | **T2D** | | **Anthropometric Traits** | | | **Glycemic Traits** | | | | | | **Blood Pressure Traits** | | **Lipids Traits** | | | | **Birth**  **Weight** |
| --- | --- | --- | --- | --- | --- | --- | --- | --- | --- | --- | --- | --- | --- | --- | --- | --- | --- | --- | --- | --- |
| **Locus** | **SNP** | **m/ M^a^** | **Exome**  **chip^b^** | **Metabo-chip^[21]^** | **BMI^[58]^** | **Height^[59]^** | **WHR^[60]^** | **FG^[4]^** | **FI^[4]^** | **HbA1c^[61]^** | **HOMA-B^[2]^** | **HOMA-IR^[2]^** | **Pro-insulin^[62]^** | **SBP^[63]^** | **DBP^[63]^** | **TG^[64]^** | **HDL-C^[64]^** | **LDL-C^[64]^** | **TC^[64]^** | **BW^[65]^** |
| *GCKR* | rs1260326 | T/**C** | C  2.4x10^-9^ | C  1.6×10^-6^ | C  0.13 | C  9.4×10^-5^ | T  0.00034 | C  2.2×10^-41^ | C  2.7×10^-22^ | C  0.31 | C  0.33 | C  9. ×10^-7^ | C  0.064 | NA  0.30 | NA  0.76 | T  2.3×10^-^ ^239^ | C  0.0017 | T  1.5×10^-7^ | T  3.1×10^-42^ | T  0.61 |
|  | rs1919128 | G/**A** | A  8.1x10^-5^ | A  0.0035 | A  0.052 | A  0.079 | G  0.0019 | NA | NA | A  0.39 | A  0.61 | A  0.0052 | A  0.07 | NA  0.073 | NA  0.35 | G  7.6×10^-104^ | A  0.16 | G  0.0036 | G  3.2×10^-19^ | A  0.27 |
|  | rs3749147 | A/**G** | G  0.00037 | NA | G  0.061 | G  0.11 | A  0.0026 | NA | NA | G  0.81 | G  0.87 | G  0.015 | G  0.048 | NA  0.12 | NA  0.48 | A  1.3×10^-53^ | G  0.075 | A  0.045 | A  3.5×10^-^ ^10^ | G  0.56 |
|  | rs1395 | **G**/A | G  0.026 | G  0.014 | G  0.72 | G  0.58 | A  0.010 | NA | NA | A  0.6 | A  0.21 | G  0.75 | G  0.67 | NA  0.23 | NA  0.66 | A  3.1×10^-38^ | G  0.57 | A  0.56 | A  2×10^-^ ^6^ | A  0.48 |
| *SLC30A8* | rs13266634 | T/**C** | C  2.5x10^-18^ | C  5×10^-21^ | T  0.024 | C  0.19 | C  0.051 | C  1.5×10^-35^ | T  0.055 | C  3.3×10^-5^ | T  2.4×10^-5^ | T  0.97 | C  4.9×10^-11^ | NA  0.44 | NA  0.57 | C  0.14 | T  0.84 | C  0.023 | C  0.054 | C  0.40 |
| *G6PC2*/ *ABCB11* | rs492594 | C/**G** **^c^** | G  0.0011 | NA | G  0.065 | C  0.27 | G  0.75 | NA | NA | C  0.25 | G  0.0026 | G  0.96 | G  0.35 | NA  0.24 | NA  0.078 | G  0.86 | C  0.063 | C  0.78 | C  0.32 | G  0.86 |
| *PCSK1* | rs6235 | G/**C** | C  0.27 | NA | G  0.0024 | G  0.0013 | C  0.66 | NA | NA | C  0.076 | G  0.024 | G  0.84 | G  7.7×10^-14^ | NA  0.43 | NA  0.12 | G  0.51 | C  0.64 | G  0.072 | G  0.085 | G  0.17 |
|  | rs6234 | C/**G** | G  0.25 | NA | C  0.0048 | C  0.0027 | G  0.40 | NA | NA | G  0.078 | C  0.041 | C  0.88 | C  1.7×10^-13^ | NA  0.58 | NA  0.24 | C  0.63 | G  0.72 | C  0.078 | C  0.086 | C  0.17 |
| *COBLL1* | rs7607980 | C/**T** | T  4.1x10^-11^ | T  2.9×10^-7^ | C  0.03 | T  0.86 | T  2.7×10^-5^ | T  0.64 | T  4.6×10^-14^ | T  0.026 | T  0.02 | T  0.017 | T  0.17 | NA  0.20 | NA  0.14 | T  2.4×10^-12^ | C  1.8×10^-15^ | T  0.29 | T  0.16 | T  0.15 |
| *TOP1* | rs17265513 | **C**/T | C  0.0012 | C  2.5×10^-6^ | T  0.41 | C  0.0098 | C  0.34 | C  8.6×10^-8^ | C  0.077 | T  0.91 | T  0.24 | C  0.49 | C  0.71 | NA  0.018 | NA  0.12 | T  0.24 | C  0.011 | C  0.00012 | C  0.00016 | T  0.15 |
| *PPARG* | rs1801282 | G/**C** | C  1.5x10^-7^ | C  1×10^-12^ | G  0.019 | G  0.037 | G  0.37 | NA | NA | C  0.59 | C  0.18 | C  0.0056 | C  0.57 | NA  0.32 | NA  0.76 | C  0.00084 | G  0.0077 | C  0.54 | C  0.84 | G  0.16 |
| *GLP1R* | rs10305492 | A/**G** | G  0.51 | NA | NA | NA | G  0.49 | NA | NA | G  0.78 | A  0.077 | A  0.71 | G  0.91 | NA  0.67 | NA  0.54 | G  0.15 | A  0.095 | G  0.31 | G  0.40 | G  0.89 |

1. Speliotes EK, Willer CJ, Berndt SI, Monda KL, Thorleifsson G, et al. (2010) Association analyses of 249,796 individuals reveal 18 new loci associated with body mass index. Nat Genet 42: 937-948.
2. Lango Allen H, Estrada K, Lettre G, Berndt SI, Weedon MN, et al. (2010) Hundreds of variants clustered in genomic loci and biological pathways affect human height. Nature 467: 832-838.
3. Heid IM, Jackson AU, Randall JC, Winkler TW, Qi L, et al. (2010) Meta-analysis identifies 13 new loci associated with waist-hip ratio and reveals sexual dimorphism in the genetic basis of fat distribution. Nat Genet 42: 949-960.
4. Soranzo N, Sanna S, Wheeler E, Gieger C, Radke D, et al. (2010) Common variants at 10 genomic loci influence hemoglobin A₁(C) levels via glycemic and nonglycemic pathways. Diabetes 59: 3229-3239.
5. Strawbridge RJ, Dupuis J, Prokopenko I, Barker A, Ahlqvist E, et al. (2011) Genome-wide association identifies nine common variants associated with fasting proinsulin levels and provides new insights into the pathophysiology of type 2 diabetes. Diabetes 60: 2624-2634.
6. Ehret GB, Munroe PB, Rice KM, Bochud M, Johnson AD, et al. (2011) Genetic variants in novel pathways influence blood pressure and cardiovascular disease risk. Nature 478: 103-109.
7. Willer CJ, Schmidt EM, Sengupta S, Peloso GM, Gustafsson S, et al. (2013) Discovery and refinement of loci associated with lipid levels. Nat Genet 45: 1274-1283.
8. Horikoshi M, Yaghootkar H, Mook-Kanamori DO, Sovio U, Taal HR, et al. (2013) New loci associated with birth weight identify genetic links between intrauterine growth and adult height and metabolism. Nat Genet 45: 76-82.
