# Supplementary material for: Identification and Functional Characterization of G6PC2 Coding Variants Influencing Glycemic Traits Define an Effector Transcript at the G6PC2-ABCB11 Locus
Source: PLoS Genet. 2015 Jan 27;11(1):e1004876. doi: 10.1371/journal.pgen.1004876 (PMC4307976; doi:10.1371/journal.pgen.1004876)
Supplement: S7 Table — m: Minor allele; M: Major allele; BMI: Body mass index; WHR: Waist-hip ratio; FG: Fasting glucose level; FI: Fasting insulin level, adjusted for BMI; HbA1c: Hemoglobin A1-C level; HOMA-B: Homeostasis model assessment-B score; HOMA-IR: Homeostasis model assessment-insulin resistance; SBP: Systolic blood pressure; DBP: Diastolic blood pressure; TG: Triglycerides; HDL-C: HDL cholesterol; LDL-C: LDL cholesterol; TC: Total cholesterol; BW: Birth weight. (a) Trait increasing allele in bold. (b) Samples contributing to T2D case-control analysis include a subset of the non-diabetic samples contributing to the current analysis. (c) All published data is “unconditioned”. As seen in our analysis, direction of effect switches after conditioning on rs560887 at least for FG. For exome-chip FG and T2D we have provided results from conditional analysis and G is the trait increasing allele. * rs2232323 (G6PC2), rs138726309 (G6PC2), rs35742417 (RREB1), and rs141203811 (URB2) have not been investigated in earlier GWAS (DOCX) [file pgen.1004876.s010.docx]

| **SNP** | **Variant** | **Minor/major allele^a^** | **MAF^b^ (%)** | **Allele counts** | **Direction of effect** | ***P*** | **Gene-based mask** |
| --- | --- | --- | --- | --- | --- | --- | --- |
| rs138726309 | p.His177Tyr | T/C | 0.78 | 502 | FG lowering | 5.98x10^-8^ | PTV + missense  PTV + NS_strict_  PTV + NS_broad_ |
| rs2232323 | p.Tyr207Ser | C/A | 0.52 | 338 | FG lowering | 9.96x10^-7^ | PTV + missense  PTV + NS_strict_  PTV + NS_broad_ |
| rs2232326 | p.Ser324Pro | C/T | 0.087 | 56 | FG lowering | 0.00044 | PTV + missense  PTV + NS_broad_ |
| rs145050507 | p.Ile171Thr | C/T | 0.13 | 81 | FG lowering | 0.0018 | PTV + missense  PTV + NS_broad_ |
| rs142189264 | p.Ser30Phe | T/C | 0.036 | 23 | FG lowering | 0.033 | PTV + missense  PTV + NS_broad_ |
| rs148689354 | p.Ile273Val | G/A | 0.0031 | 2 | FG increasing | 0.052 | PTV + missense |
| rs199682245 | p.Asn68Ile | T/A | 0.0031 | 2 | FG lowering | 0.12 | PTV + missense  PTV + NS_strict_  PTV + NS_broad_ |
| rs145217135 | p.Ile230Thr | C/T | 0.022 | 14 | FG lowering | 0.12 | PTV + missense  PTV + NS_broad_ |
| rs150538801 | p.Phe256Leu | C/T | 0.014 | 9 | FG increasing | 0.32 | PTV + missense  PTV + NS_broad_ |
| rs201561079 | p.Ile63Thr | C/T | 0.0093 | 6 | FG lowering | 0.34 | PTV + missense  PTV + NS_broad_ |
| rs200336133 | p.Leu310Phe | T/C | 0.065 | 42 | FG increasing | 0.44 | PTV + missense |
| rs2232322 | p.Ile171Val | G/A | 0.0062 | 4 | FG increasing | 0.54 | PTV + missense  PTV + NS_broad_ |
| rs146779637 | p.Arg283* | T/C | 0.11 | 71 | FG lowering | 0.67 | PTV-only  PTV + NS_strict_ |
| rs149874491 | p.Ile38Leu | C/A | 0.0016 | 1 | FG increasing | 0.91 | PTV + missense |
| rs184807114 | p.Ala119Val | T/C | 0.036 | 23 | FG lowering | 0.97 | PTV + missense  PTV + NS_broad_ |
