# Supplementary material for: Identification and Functional Characterization of G6PC2 Coding Variants Influencing Glycemic Traits Define an Effector Transcript at the G6PC2-ABCB11 Locus
Source: PLoS Genet. 2015 Jan 27;11(1):e1004876. doi: 10.1371/journal.pgen.1004876 (PMC4307976; doi:10.1371/journal.pgen.1004876)
Supplement: S8 Table — N: Number of samples. (DOCX) [file pgen.1004876.s011.docx]

| SNP | N | Genotype counts | | Genotype concordance | |
| --- | --- | --- | --- | --- | --- |
|  |  | Heterozygous | Non-reference homozygous | Heterozygous | Non-reference homozygous |
| rs141203811 | 3999 | 6 | 0 | 1 | NA |
| rs1395 | 3998 | 1640 | 1091 | 1 | 1 |
| rs1260326 | 3998 | 1780 | 1081 | 1 | 1 |
| rs1919128 | 2312 | 922 | 159 | 1 | 1 |
| rs3749147 | 3995 | 1422 | 221 | 0.998 | 1 |
| rs7607980 | 4000 | 769 | 48 | 1 | 1 |
| rs142189264 | 4000 | 4 | 0 | 1 | NA |
| rs201561079 | 3998 | 4 | 0 | 0.750 | NA |
| rs145050507 | 4000 | 12 | 0 | 1 | NA |
| rs138726309 | 4000 | 50 | 0 | 1 | NA |
| rs2232323 | 4000 | 43 | 0 | 1 | NA |
| rs492594 | 3999 | 1964 | 912 | 1 | 1 |
| rs145217135 | 3414 | 1 | 0 | 1 | NA |
| rs150538801 | 3999 | 1 | 0 | 1 | NA |
| rs146779637 | 4000 | 12 | 0 | 1 | NA |
| rs200336133 | 2312 | 4 | 0 | 1 | NA |
| rs2232326 | 4000 | 13 | 0 | 1 | NA |
| rs1801282 | 4000 | 955 | 83 | 1 | 1 |
| rs6235 | 3999 | 1635 | 341 | 1 | 1 |
| rs6234 | 3999 | 1636 | 341 | 1 | 1 |
| rs35742417 | 3412 | 1147 | 138 | 1 | 0.986 |
| rs10305492 | 4000 | 129 | 1 | 1 | 1 |
| rs13266634 | 4000 | 1754 | 496 | 1 | 1 |
| rs17265513 | 3999 | 1460 | 242 | 1 | 1 |
