# Supplementary material for: Identification and Functional Characterization of G6PC2 Coding Variants Influencing Glycemic Traits Define an Effector Transcript at the G6PC2-ABCB11 Locus
Source: PLoS Genet. 2015 Jan 27;11(1):e1004876. doi: 10.1371/journal.pgen.1004876 (PMC4307976; doi:10.1371/journal.pgen.1004876)
Supplement: S9 Table — CR: call rate. HWE: Hardy-Weinberg equilibrium p-value. (DOCX) [file pgen.1004876.s012.docx]

| **SNP** | **FIN-D2D 2007** | | **The Finnish Diabetes Prevention Study (DPS)** | | **The Dose Responses to Exercise Training (DR's EXTRA) Study** | | **National FINRISK 2007 Study (FINRISK 2007)** | | **Finland-United States Investigation of NIDDM Genetics (FUSION) Study** | | **Metabolic Syndrome in Men Study (METSIM)** | | **Health2006** | | **Inter99** | | **Vejle Biobank** | | **Genetics of Diabetes Audit and Research Tayside (GoDARTS)** | | **Twins UK** | | **Oxford BioBank (OBB)** | | **Prospective Investigation of the Vasculature in Uppsala Seniors (PIVUS)** | | **Uppsala Longitudinal Study of Adult Men (ULSAM)** | | **Prevalence, Prediction and Prevention of Diabetes (PPP)-Botnia study** | |
| --- | --- | --- | --- | --- | --- | --- | --- | --- | --- | --- | --- | --- | --- | --- | --- | --- | --- | --- | --- | --- | --- | --- | --- | --- | --- | --- | --- | --- | --- | --- |
|  | CR | HWE | CR | HWE | CR | HWE | CR | HWE | CR | HWE | CR | HWE | CR | HWE | CR | HWE | CR | HWE | CR | HWE | CR | HWE | CR | HWE | CR | HWE | CR | HWE | CR | HWE |
| rs141203811 | 1.00 | 1.00 | 1.00 | 1.00 | 1.00 | 1.00 | 1.00 | 1.00 | 1.00 | 1.00 | 1.00 | 1.00 | 1.00 | 1.00 | 1.00 | 1.00 | 1.00 | 1.00 | 1.00 | 1.00 | 1.00 | 1.00 | 1.00 | 1.00 | 1.00 | 1.00 | 1.00 | 1.00 | 1.00 | NA |
| rs1395 | 1.00 | 0.11 | 1.00 | 0.89 | 1.00 | 0.07 | 1.00 | 1.00 | 1.00 | 0.03 | 1.00 | 0.69 | 1.00 | 0.16 | 1.00 | 0.16 | 1.00 | 0.16 | 1.00 | 0.97 | 1.00 | 0.97 | 1.00 | 0.97 | 1.00 | 0.30 | 1.00 | 0.30 | 1.00 | 0.34 |
| rs1260326 | 1.00 | 0.54 | 1.00 | 0.08 | 1.00 | 0.81 | 1.00 | 0.12 | 1.00 | 0.12 | 1.00 | 0.64 | 1.00 | 0.10 | 1.00 | 0.10 | 1.00 | 0.10 | 1.00 | 1.00 | 1.00 | 1.00 | 1.00 | 1.00 | 1.00 | 0.26 | 1.00 | 0.26 | 1.00 | 0.43 |
| rs1919128 | 1.00 | 0.41 | 1.00 | 0.79 | 1.00 | 0.79 | 1.00 | 0.61 | 1.00 | 0.15 | 1.00 | 0.68 | 1.00 | 0.91 | 1.00 | 0.91 | 1.00 | 0.91 | 1.00 | 0.40 | 1.00 | 0.40 | 1.00 | 0.40 | 1.00 | 0.90 | 1.00 | 0.90 | 1.00 | 0.55 |
| rs3749147 | 0.99 | 0.33 | 1.00 | 0.56 | 1.00 | 0.25 | 0.99 | 0.91 | 0.99 | 0.17 | 1.00 | 1.00 | 1.00 | 0.93 | 1.00 | 0.93 | 1.00 | 0.93 | 1.00 | 0.60 | 1.00 | 0.60 | 1.00 | 0.60 | 1.00 | 0.70 | 1.00 | 0.70 | 1.00 | NA |
| rs7607980 | 1.00 | 0.19 | 1.00 | 0.24 | 1.00 | 1.00 | 1.00 | 0.68 | 1.00 | 0.66 | 1.00 | 0.71 | 1.00 | 0.74 | 1.00 | 0.74 | 1.00 | 0.74 | 1.00 | 0.62 | 1.00 | 0.62 | 1.00 | 0.62 | 1.00 | 0.23 | 1.00 | 0.23 | 1.00 | 0.38 |
| rs142189264 | 1.00 | 1.00 | 1.00 | 1.00 | 1.00 | 1.00 | 1.00 | 1.00 | 1.00 | 1.00 | 1.00 | 1.00 | 1.00 | 1.00 | 1.00 | 1.00 | 1.00 | 1.00 | 1.00 | 1.00 | 1.00 | 1.00 | 1.00 | 1.00 | 1.00 | 1.00 | 1.00 | 1.00 | 1.00 | NA |
| rs149874491 | 1.00 | 1.00 | 1.00 | 1.00 | 1.00 | 1.00 | 1.00 | 1.00 | 1.00 | 1.00 | 1.00 | 1.00 | 1.00 | 1.00 | 1.00 | 1.00 | 1.00 | 1.00 | 1.00 | 1.00 | 1.00 | 1.00 | 1.00 | 1.00 | 1.00 | 1.00 | 1.00 | 1.00 | 1.00 | NA |
| rs201561079 | 1.00 | 1.00 | 1.00 | 1.00 | 1.00 | 1.00 | 1.00 | 1.00 | 1.00 | 1.00 | 1.00 | 1.00 | 1.00 | 1.00 | 1.00 | 1.00 | 1.00 | 1.00 | 1.00 | 1.00 | 1.00 | 1.00 | 1.00 | 1.00 | 1.00 | 1.00 | 1.00 | 1.00 | 1.00 | 1.00 |
| rs199682245 | 1.00 | 1.00 | 1.00 | 1.00 | 1.00 | 1.00 | 1.00 | 1.00 | 1.00 | 1.00 | 1.00 | 1.00 | NA | NA | NA | NA | NA | NA | 1.00 | 1.00 | 1.00 | 1.00 | 1.00 | 1.00 | 1.00 | 1.00 | 1.00 | 1.00 | 1.00 | NA |
| rs184807114 | 1.00 | 1.00 | 1.00 | 1.00 | 1.00 | 1.00 | 1.00 | 1.00 | 1.00 | 1.00 | 1.00 | 1.00 | 1.00 | 1.00 | 1.00 | 1.00 | 1.00 | 1.00 | 1.00 | 1.00 | 1.00 | 1.00 | 1.00 | 1.00 | 1.00 | 1.00 | 1.00 | 1.00 | 1.00 | NA |
| rs2232322 | 1.00 | 1.00 | 1.00 | 1.00 | 1.00 | 1.00 | 1.00 | 1.00 | 1.00 | 1.00 | 1.00 | 1.00 | 1.00 | 1.00 | 1.00 | 1.00 | 1.00 | 1.00 | 1.00 | 1.00 | 1.00 | 1.00 | 1.00 | 1.00 | 1.00 | 1.00 | 1.00 | 1.00 | 1.00 | NA |
| rs145050507 | 1.00 | 1.00 | 1.00 | 1.00 | 1.00 | 1.00 | 1.00 | 1.00 | 1.00 | 1.00 | 1.00 | 1.00 | 1.00 | 1.00 | 1.00 | 1.00 | 1.00 | 1.00 | 1.00 | 1.00 | 1.00 | 1.00 | 1.00 | 1.00 | 1.00 | 1.00 | 1.00 | 1.00 | 1.00 | 1.00 |
| rs138726309 | 1.00 | 1.00 | 1.00 | 1.00 | 1.00 | 1.00 | 1.00 | 0.11 | 1.00 | 1.00 | 1.00 | 1.00 | 1.00 | 0.53 | 1.00 | 0.53 | 1.00 | 0.53 | 1.00 | 1.00 | 1.00 | 1.00 | 1.00 | 1.00 | 1.00 | 1.00 | 1.00 | 1.00 | 1.00 | 1.00 |
| rs2232323 | 1.00 | 1.00 | 1.00 | 1.00 | 1.00 | 1.00 | 1.00 | 1.00 | 1.00 | 1.00 | 1.00 | 1.00 | 1.00 | 0.63 | 1.00 | 0.63 | 1.00 | 0.63 | 1.00 | 0.45 | 1.00 | 0.45 | 1.00 | 0.45 | 1.00 | 1.00 | 1.00 | 1.00 | 1.00 | 1.00 |
| rs492594 | 1.00 | 0.54 | 1.00 | 0.27 | 1.00 | 0.58 | 1.00 | 1.00 | 1.00 | 0.49 | 1.00 | 0.24 | 1.00 | 0.84 | 1.00 | 0.84 | 1.00 | 0.84 | 1.00 | 0.21 | 1.00 | 0.21 | 1.00 | 0.21 | 1.00 | 0.66 | 1.00 | 0.66 | 1.00 | 0.78 |
| rs145217135 | 1.00 | 1.00 | 1.00 | 1.00 | 1.00 | 1.00 | 1.00 | 1.00 | 1.00 | 1.00 | 1.00 | 1.00 | 1.00 | 1.00 | 1.00 | 1.00 | 1.00 | 1.00 | 1.00 | 1.00 | 1.00 | 1.00 | 1.00 | 1.00 | 1.00 | 1.00 | 1.00 | 1.00 | 1.00 | NA |
| rs150538801 | 1.00 | 1.00 | 1.00 | 1.00 | 1.00 | 1.00 | 1.00 | 1.00 | 1.00 | 1.00 | 1.00 | 1.00 | 1.00 | 1.00 | 1.00 | 1.00 | 1.00 | 1.00 | 1.00 | NA | 1.00 | NA | 1.00 | NA | 1.00 | NA | 1.00 | NA | 1.00 | 1.00 |
| rs148689354 | 1.00 | 1.00 | 1.00 | 1.00 | 1.00 | 1.00 | 1.00 | 1.00 | 1.00 | 1.00 | 1.00 | 1.00 | 1.00 | 1.00 | 1.00 | 1.00 | 1.00 | 1.00 | 1.00 | 1.00 | 1.00 | 1.00 | 1.00 | 1.00 | 1.00 | 1.00 | 1.00 | 1.00 | 1.00 | NA |
| rs146779637 | 1.00 | 1.00 | 1.00 | 1.00 | 1.00 | 1.00 | 1.00 | 1.00 | 1.00 | 1.00 | 1.00 | 1.00 | 1.00 | 1.00 | 1.00 | 1.00 | 1.00 | 1.00 | 1.00 | 1.00 | 1.00 | 1.00 | 1.00 | 1.00 | 1.00 | 1.00 | 1.00 | 1.00 | 1.00 | 1.00 |
| rs200336133 | 1.00 | 1.00 | 1.00 | 1.00 | 1.00 | 1.00 | 1.00 | 1.00 | 1.00 | 1.00 | 1.00 | 1.00 | 1.00 | 1.00 | 1.00 | 1.00 | 1.00 | 1.00 | 1.00 | 1.00 | 1.00 | 1.00 | 1.00 | 1.00 | 1.00 | 1.00 | 1.00 | 1.00 | 1.00 | 0.08 |
| rs2232326 | 1.00 | 1.00 | 1.00 | 1.00 | 1.00 | 1.00 | 1.00 | 1.00 | 1.00 | 1.00 | 1.00 | 1.00 | 1.00 | 1.00 | 1.00 | 1.00 | 1.00 | 1.00 | 1.00 | 0.09 | 1.00 | 0.09 | 1.00 | 0.09 | 1.00 | 1.00 | 1.00 | 1.00 | 1.00 | 1.00 |
| rs1801282 | 1.00 | 0.22 | 1.00 | 1.00 | 1.00 | 0.65 | 1.00 | 0.77 | 1.00 | 0.47 | 1.00 | 1.00 | 1.00 | 0.77 | 1.00 | 0.77 | 1.00 | 0.77 | 1.00 | 0.86 | 1.00 | 0.86 | 1.00 | 0.86 | 1.00 | 0.38 | 1.00 | 0.38 | 1.00 | 0.21 |
| rs6235 | 1.00 | 0.16 | 1.00 | 0.18 | 1.00 | 0.06 | 1.00 | 0.75 | 1.00 | 0.31 | 1.00 | 0.51 | 1.00 | 0.28 | 1.00 | 0.28 | 1.00 | 0.28 | 1.00 | 0.45 | 1.00 | 0.45 | 1.00 | 0.45 | 1.00 | 1.00 | 1.00 | 1.00 | 1.00 | 0.40 |
| rs6234 | 1.00 | 0.16 | 1.00 | 0.18 | 1.00 | 0.06 | 1.00 | 0.83 | 1.00 | 0.31 | 1.00 | 0.49 | 1.00 | 0.24 | 1.00 | 0.24 | 1.00 | 0.24 | 1.00 | 0.37 | 1.00 | 0.37 | 1.00 | 0.37 | 1.00 | 1.00 | 1.00 | 1.00 | 1.00 | 0.40 |
| rs35742417 | 1.00 | 1.00 | 1.00 | 0.62 | 1.00 | 0.02 | 1.00 | 0.31 | 1.00 | 1.00 | 1.00 | 0.69 | 1.00 | 0.18 | 1.00 | 0.18 | 1.00 | 0.18 | 1.00 | 0.49 | 1.00 | 0.49 | 1.00 | 0.49 | 1.00 | 0.65 | 1.00 | 0.65 | 1.00 | 0.68 |
| rs10305492 | 1.00 | 1.00 | 1.00 | 1.00 | 1.00 | 1.00 | 1.00 | 1.00 | 1.00 | 1.00 | 1.00 | 1.00 | 1.00 | 0.82 | 1.00 | 0.82 | 1.00 | 0.82 | 1.00 | 0.41 | 1.00 | 0.41 | 1.00 | 0.41 | 1.00 | 0.48 | 1.00 | 0.48 | 1.00 | 0.04 |
| rs13266634 | 1.00 | 0.31 | 1.00 | 1.00 | 1.00 | 0.81 | 1.00 | 0.86 | 1.00 | 0.82 | 1.00 | 0.29 | 1.00 | 0.35 | 1.00 | 0.35 | 1.00 | 0.35 | 1.00 | 0.34 | 1.00 | 0.34 | 1.00 | 0.34 | 1.00 | 0.42 | 1.00 | 0.42 | 1.00 | 0.13 |
| rs17265513 | 1.00 | 0.07 | 1.00 | 0.61 | 1.00 | 1.00 | 1.00 | 0.35 | 1.00 | 0.23 | 1.00 | 0.40 | 1.00 | 0.83 | 1.00 | 0.83 | 1.00 | 0.83 | 1.00 | 0.87 | 1.00 | 0.87 | 1.00 | 0.87 | 1.00 | 0.85 | 1.00 | 0.85 | 1.00 | 0.83 |
